# Health belief model-based educational interventions for knowledge, beliefs, and intentions on mammography: a systematic review

**DOI:** 10.1186/s12905-025-04218-9

**Published:** 2025-12-22

**Authors:** Ahmad Shaker Abu Abed, Luz Garcia-Valdes, Hana Taha, Carmen Amezcua-Prieto

**Affiliations:** 1 Department of Preventive Medicine and Public Health, Faculty of Medicine, University of Granada, Granada, Spain; 2 Department of Family and Community Medicine, School of Medicine, The University of Jordan, Amman, Jordan; 3 Department of Neurobiology, Care Sciences and Society, Karolinska Institute, Stockholm, Sweden; 4 CIBER of Epidemiology and Public Health, Carlos III Health Institute, Madrid, Spain; 5 Instituto de Investigación Biosanitaria ibs, Granada, Spain

**Keywords:** Mammography, Attitudes, Practice, Health belief model, Health education interventions, Women´s health, Breast cancer

## Abstract

**Background:**

Breast cancer (BC) is a significant global health issue and the most common cancer among women. Early detection via mammography is crucial for improving survival rates. This systematic review (SR) explores the impact of educational interventions based on the Health Belief Model (HBM) on women’s BC knowledge, beliefs, and intentions regarding mammography among women aged 40 and older.lease be informed that I submitted

**Methods:**

The SR was registered on PROSPERO (CRD42023402436) and followed the Preferred Reporting Items for Systematic Reviews and Meta-Analyses (PRISMA, 2020) guidelines. A comprehensive search was conducted across five databases—PubMed, CINAHL, Embase, Web of Science, and PsycINFO—for relevant English-language studies published from January 2003 to December 2024. The study quality was assessed using the Cochrane Risk of Bias 2 (RoB 2) and the Revised Risk of Bias Assessment 2 (RoBANS 2) tools. A narrative synthesis was conducted following established methodological guidance.

**Results:**

Eight studies were included, consisting of five randomized controlled trials (RCTs) and three non-randomized controlled trials (NRCTs), with a total of 1,439 participants. The interventions included individual, group, and multimedia education and consultations. Six studies showed significant improvement in knowledge, while seven showed improvement in one or more constructs of the CHBMS related to beliefs about mammogram screening. Key factors influencing screening intentions were embarrassment, cost, income level, health insurance, age, and immigration status. Limitations of the studies included small sample sizes, reliance on self-reported data, lack of control groups, and short follow-up periods.

**Conclusion:**

Educational interventions based on HBM generally improve BC knowledge, beliefs, and intentions about mammography in women aged 40 years and older. Interventions that incorporate multiple strategies within healthcare settings show the most significant improvements. Future approaches should be multifaceted, sensitive to cultural and socioeconomic contexts, and include ongoing follow-up to promote screening adherence and early BC detection.

**Supplementary Information:**

The online version contains supplementary material available at 10.1186/s12905-025-04218-9.

## Background

Breast cancer is the most diagnosed cancer among women and a leading cause of cancer-related deaths globally. In 2022, there were 2,296,840 cases, accounting for 11.5% of new cancer diagnoses and around 670,000 deaths, making it the fourth leading cause of cancer death [1]. Its rising incidence and high treatment costs challenge healthcare systems worldwide [1, 2]. Early detection is crucial for improving BC prognosis through screenings such as mammography, clinical breast examination (CBE), and breast self-examination (BSE) [3, 4]. Although BSE is simple and cost-effective, it has limited diagnostic efficacy and is no longer recommended as a standalone method [4, 5]. Both BSE and CBE are insufficient for definitive diagnosis and mainly detect palpable, symptomatic lumps [4–6]. In contrast, mammography effectively screens asymptomatic women and can detect tumors one to two years earlier, making it the preferred method for early detection [6–10].

In the United States (U.S.), BC incidence is significantly higher in women over 40, who account for most diagnoses, while those under 40 make up only 4% of cases [4, 11]. Consequently, prominent health organizations such as the National Comprehensive Cancer Network (NCCN) and the American College of Radiology (ACR) emphasize the need for regular mammography screening among women aged 40 years and older [3, 12, 13]. Despite global awareness campaigns, adherence to breast cancer screening (BCS) guidelines remains low [14–16]. Evidence indicates that merely raising awareness through education is insufficient to alter behavior; addressing deep-rooted beliefs and attitudes shaped by cultural, religious, and personal factors is essential yet challenging [6, 17, 18].

Behavioral health theories are crucial for understanding health behaviors. The HBM is a leading framework for designing interventions and explaining behavior change [9, 19]. It effectively predicts intentions to undergo screening and increases rates of mammography uptake [20, 21]. While other theories, such as the Theory of Reasoned Action, offer insights through subjective norms, the HBM’s focus on perceptions makes it preferable for health promotion programs [22, 23]. Various interventions have been developed to enhance BCS participation, emphasizing educational strategies and culturally tailored approaches. SRs show that these initiatives significantly improve screening rates and enhance knowledge, especially in underserved populations [24–26]. The HBM influences screening behaviors by addressing perceived susceptibility, severity, benefits, and barriers [27, 28]. Additionally, tailored interventions have proven to be more effective than generic ones [29, 30].

Despite advancements in BCS methods, considerable challenges remain, including inadequate healthcare infrastructure, socioeconomic disparities, and cultural stigma that hinder access to screening services [16, 31, 32]. Concerns about the sustainability of these interventions and the lack of strategies addressing systemic health determinants are prevalent [3, 33]. The inconsistent integration of behavioral theories and digital technologies further restricts their scalability [26, 34]. Given the rise in late-stage breast cancer diagnoses [14], ongoing research seeks effective prevention strategies. While previous research has suggested educational interventions to improve screening rates [11, 35], many of these studies face methodological limitations, including inconsistent theoretical application, unclear participant demographics, and a narrow focus on specific populations [25, 26]. This SR aims to explore the impact and identify key factors within HBM-based educational programs’ effects on women’s knowledge, beliefs, and intentions regarding mammograms among women aged 40 years and older.

### Systematic review questions

1. What are the factors that influence the outcomes of HBM-based educational interventions on mammography screening among women aged 40 years and older?
2. What are the reported effects of HBM-based educational interventions on BC knowledge, beliefs, and intentions regarding mammography screening among women aged 40 years and older?
3. What are the recent and effective methodologies that have been employed in HBM-based educational interventions to address mammography screening among women aged 40 years and older?

## Materials and methods

This SR was conducted following the population, intervention, comparator, outcome, and study design (PICOS) framework and registered in the International Prospective Register of Systematic Reviews (PROSPERO) under the number CRD42023402436 [36]. The study adhered to the Preferred PRISMA 2020 guidelines for study design [37] (Supplementary Checklists 1 and 2).

### Conceptual and analytic framework

The HBM is a theoretical framework that explains individuals’ preventive health behaviors in various contexts, such as vaccination and smoking cessation. It suggests that motivation to participate in health-promoting actions is influenced by individuals’ perceptions of their susceptibility to health issues, the severity of those issues, and the benefits of taking action [9, 19]. The HBM marks a shift from traditional biomedical approaches by emphasizing the psychological factors involved in health decision-making [19, 23]. Unlike some other behavioral theories, the HBM effectively tailors interventions based on personal beliefs, leading to improved screening behaviors [9, 19, 23]. While the Theory of Planned Behavior (TPB) and Social Cognitive Theory (SCT) focus on attitudes and self-efficacy, they may not address specific health beliefs as well as the HBM does. Broader models, such as the Transtheoretical Model and the Ecological Model, offer broader perspectives but lack the emphasis on individual perceptions that the HBM offers [9, 38, 39]. Overall, the HBM is essential for promoting health, preventing disease, and informing public health interventions [19] (Supplementary Fig. 1).

Victoria Champion developed the Champion Health Belief Model (CHBM) as an adaptation of the original HBM better to understand women’s participation in BCS [20, 40]. Champion initially measured the HBM constructs for BSE [20, 40]. This research later evolved into the creation of CHBMS for BSE and mammography [20, 40]. The tool emphasizes health motivation and self-efficacy while considering cultural factors [20]. The tool has been translated and validated in various studies in countries such as Jordan, Turkey, Qatar, and Nigeria [31, 41–43]. Ultimately, this SR is guided by the HBM, while the theoretical assumptions for the data outcomes and analysis were based on CHMBS.

### Search strategy

The search and selection of studies in this SR were conducted using EndNote citation management software, version 21, which facilitated the importing of relevant studies sourced from five databases: PubMed, CINAHL, Embase, Web of Science, and PsycINFO. The search focused on studies published between 2003 and 2024, guided by the PICOS framework, which guided both the formulation of the research question and the eligibility criteria for the included studies. The (P): women aged 40 years and older. (I): educational interventions based on the HBM; (C): included control groups with no intervention, usual care, or alternative interventions, along with single-group pre-post studies; (O): outcomes were measurable changes in knowledge, beliefs aligned with CHBMS constructs, and intentions regarding mammogram screening, (S): RCTs or NRCTs studies.

**Fig. 1:**
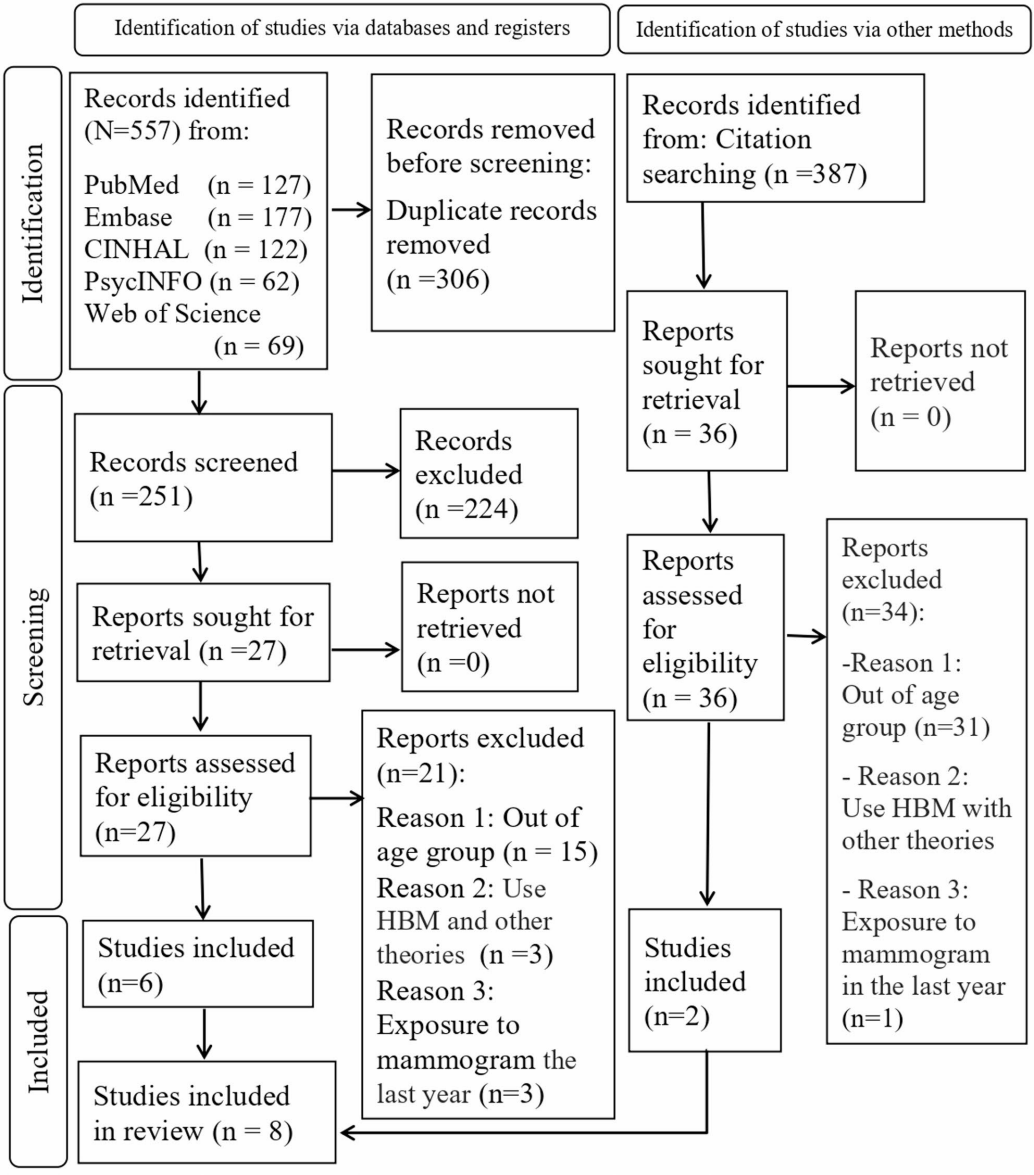
PRISMA 2020 flow diagram of the review study selection process.Footnote:Source: Page MJ, McKenzie JE, Bossuyt PM, et al. The PRISMA 2020 statement: an updated guideline for reporting systematic reviews. BMJ. 2021;372:n71. doi:10.1136/bmj.n71

The eligibility criteria for this SR were explicitly designed to select studies that aligned with its objective and questions. The inclusion criteria required RCTs or NRCTs featuring HBM-based educational interventions, involving women aged 40 and older who had not received a mammogram in the last year and had no history of BC, or a first-degree relative with BC. The search was limited to English-language studies published between 2003 and 2024 across five databases. Studies were excluded if they did not use the CHBMS and a knowledge questionnaire for outcomes, if mammography was not part of the intervention, or if the HBM was not the primary theoretical foundation for the intervention. The PICOS framework guided the development of the Medical Subject Headings (MeSH) using PubMed’s MeSH Builder. Refined by the Yale MeSH Analyzer and MeSH browser online tools. The search strategy was optimized using PubMed’s Advanced Search Builder, which included mainly “Breast Neoplasm,” “Health Belief Model,” “Mammography,” “Education,” “Health Knowledge, Attitudes, Practice,” and “Women,” and incorporated free-text keywords. After finalizing the PubMed search syntax, it was adapted for other databases, such as Embase, using Emtree terms. This structured approach ensured a systematic search and selection (Supplementary Table 1). Two authors, AA and LGV, conducted the screening process at two time points: an initial screening in April 2023 and a follow-up in December 2024, selecting relevant studies based on eligibility criteria. After removing duplicates, they reviewed titles and abstracts, resolving disagreements through consensus or consultation with a third author, CAP. Full texts were assessed independently by AA and LGV, with discrepancies addressed by CAP. An automated citation chaser tool was used for backward and forward reference searches to ensure thorough coverage (Fig. 1) [44].

### Data extraction

Data extraction from the included studies was conducted independently by two authors, AA and LGV, using a pre-designed Excel table that aligned with study objectives. Any disagreements were resolved through consensus or with a third author, HT. The data extraction focused on three main outcomes: (1) BC Knowledge; (2) Health beliefs assessed through the CHBMS constructs (Perceived susceptibility, severity, benefits, barriers, health motivation, and self-efficacy); and (3) Behavioral intentions. Furthermore, information regarding the interventional methodologies and the sociodemographic characteristics was systematically collected. The synthesis of data was executed narratively.

The narrative synthesis was conducted following the guidance outlined by Popay et al. (2006) [45]. This process involved several steps: (1) Developing a theory regarding how the interventions work, their rationale, and their target populations; (2) Creating a preliminary synthesis of findings; (3) Examining relationships within the data; and (4) Evaluating the robustness of the synthesis. To achieve this, we tabulated the characteristics and results of the studies, grouped them by intervention type and population, and thematically analyzed the factors that influenced outcomes. We did not perform any new statistical analyses, such as a meta-analysis; all p-values and statistical results reported are those presented in the original studies included in the review.

### Quality assessment

The quality assessment was conducted by two authors, AA and LGV. The Cochrane RoB 2 tool was used for the RCTs, and the RoBANS 2 tool for the NRCTs. Following the assessment of each study, the authors discussed their findings and addressed any discrepancies by either reaching consensus or consulting the third author, HT.

## Results

### Study selection

In the initial and follow-up searches conducted in 2023 and 2024, a total of 557 articles were identified from five databases and imported into EndNote. After removing 306 duplicates, 251 unique articles were screened by title and abstract, and 224 were excluded for not meeting the inclusion criteria. This left 27 articles for full-text review, from which 21 were excluded, resulting in six studies included [46–51]. Using the CitationChaser online tool, 387 additional studies were traced, of which two were relevant [52, 53]. This led to a total of eight studies included in this SR, as illustrated in the PRISMA flow diagram (Fig. 1, Supplementary Table 2).

### Quality assessment

Quality assessment using the RoB 2 and RoBANS 2 tools revealed that seven of the eight included studies demonstrated a low risk of bias [46–48, 50, 52, 54, 55]. Among the five RCTs, four studies [46, 47, 50, 52] exhibited low risk in most domains, indicating robust randomization, complete outcome data, and a low risk of selective reporting. One RCT was assessed as having “Some Concerns” due to the lack of blinding for participants and personnel [49]. The three NRCTs were assessed as having a low risk of bias [48, 54, 55]. However, unclear blinding of outcome assessors and the inherent limitations of non-randomized designs may affect the internal validity (Supplementary Tables 3–6).

### Included studies characteristics

This SR included eight studies involving 1,439 women aged 40 years and older. Sample sizes varied across the studies, ranging from 43 to 327 participants, and the studies were conducted in three regions: the U.S [52, 54, 55]., Iran [47, 49, 50], and Turkey [46, 48]. The interventions took place in diverse settings, including community centers [48, 52, 54, 55], educational institutions [49], and healthcare facilities [46, 47, 50]. Most participants in the studies were married [46–52]. Furthermore, three studies specifically focused on immigrant women in the U.S [52, 54, 55]. (Supplementary Table 7).

### Interventions characteristics

All studies incorporated pre-test and post-test questionnaires, with follow-up periods ranging from immediately after the intervention to six months later. Data were collected via questionnaires, either face-to-face [46, 48–50, 54] or remotely [47, 52, 55]. A variety of techniques were employed for delivering the educational interventions, with seven studies adopting multifaceted strategies, including group education, consultations, printed materials, and multimedia resources [46–50, 52, 54]. All studies featured either an intervention and control group or multiple intervention groups, except for two NRCTs [54, 55]. Furthermore, the NRCTs utilized convenience sampling [48, 54, 55]. Due to variability in methodologies and outcomes, a meta-analysis was not feasible (Table 1and 2).

**Table 1:** Methodology and the intervention characteristics of the included studies

Author/ Year	Study Design, Sampling Method	Data Collection Methods, Follow-up Period	Intervention	Control
Garza/ 2005	NRCT Convenience sampling	Pretest and Post-test Questionnaire (Face to Face), Three months	The modified series design involved four stages: - In the first stage, Community health workers were recruited and trained to conduct home visits. Participants were given two brochures. - Second stage, home visits and questionnaire - Third stage, a 2-hour educational intervention. - The fourth stage, scheduling an appointment.	No Control
Wang/ 2008	NRCT Convenience sampling,	Pretest and Post-test Questionnaire (Phone), Immediate	The study was conducted in three phases. -Formative phase: to format a video. -Production phase: to produce a video. -Quantitative phase: Participants watched a 17-minute tailored Chinese video.	No Control
Secginli & Nahcivan/ 2011	RCT, Random Sampling	Pretest and Post-test Questionnaire (Face to Face), Immediate, after 3 and 6 months	The intervention group received a 120-minute breast health promotion program that included: 1. Breast health education, a teaching session to small groups of 5 to 8 women. The flip chart used pages with graphics. 2. Film, booklet, calendar, and a card.	General information that excludes breast health.
Rezaeian/ 2014	Population-RCT, Random Sampling	Pretest and Post-test Questionnaire (Face to Face), Four months	The educational program consists of four educational sessions, each session lasts 90 min, covered various topics, and used different teaching methods. - Two pamphlets.	No intervention, Routine, or normal life
Seven/ 2015	NRCT, Convenience Sampling with Two Stratification Methods	Pretest and Post-test Questionnaire (Face to Face), Three months	Participants were divided into one of three educational groups: as follows: - Individual one-on-one education and brochure, - Individual one-on-one education and brochure designed for women, and another for spouses. - Group Education: attended educational sessions lasting 60–90 min, and a brochure.	No Control
Heydari & Noroozi/ 2015	RCT, Random Sampling	Pretest and Post-test Questionnaire (Face to Face), Three months	Two intervention groups that received either a - Group education, the participants were trained in two sessions of 45 to 60 min. - Multimedia education involves the same educational materials, but it is delivered through SMS and CDs.	No Control
Wu & Lin/2015	RCT, Single-Blind Study, Random Sampling	Pretest and Post-test Questionnaire (Phone), Four months	Web-based, individually tailored program developed for the telephone counselling. - The intervention group received a tailored, personalized intervention based on the results of their baseline interviews. They were also received a counselling messages tailored to these results.	Received a mammography pamphlet on breast health
Mirmoammadi/2018	RCT, Two Steps cluster, Random Sampling	Pretest and Post-test Questionnaire (Face to Face), Three months	The intervention group received: - Four educational sessions, one session of 90 min per week for four weeks, including group discussion, practical training, a training booklet, a test, and Individual consultations.	Received routine care from the health care centers.

**Table 2:**
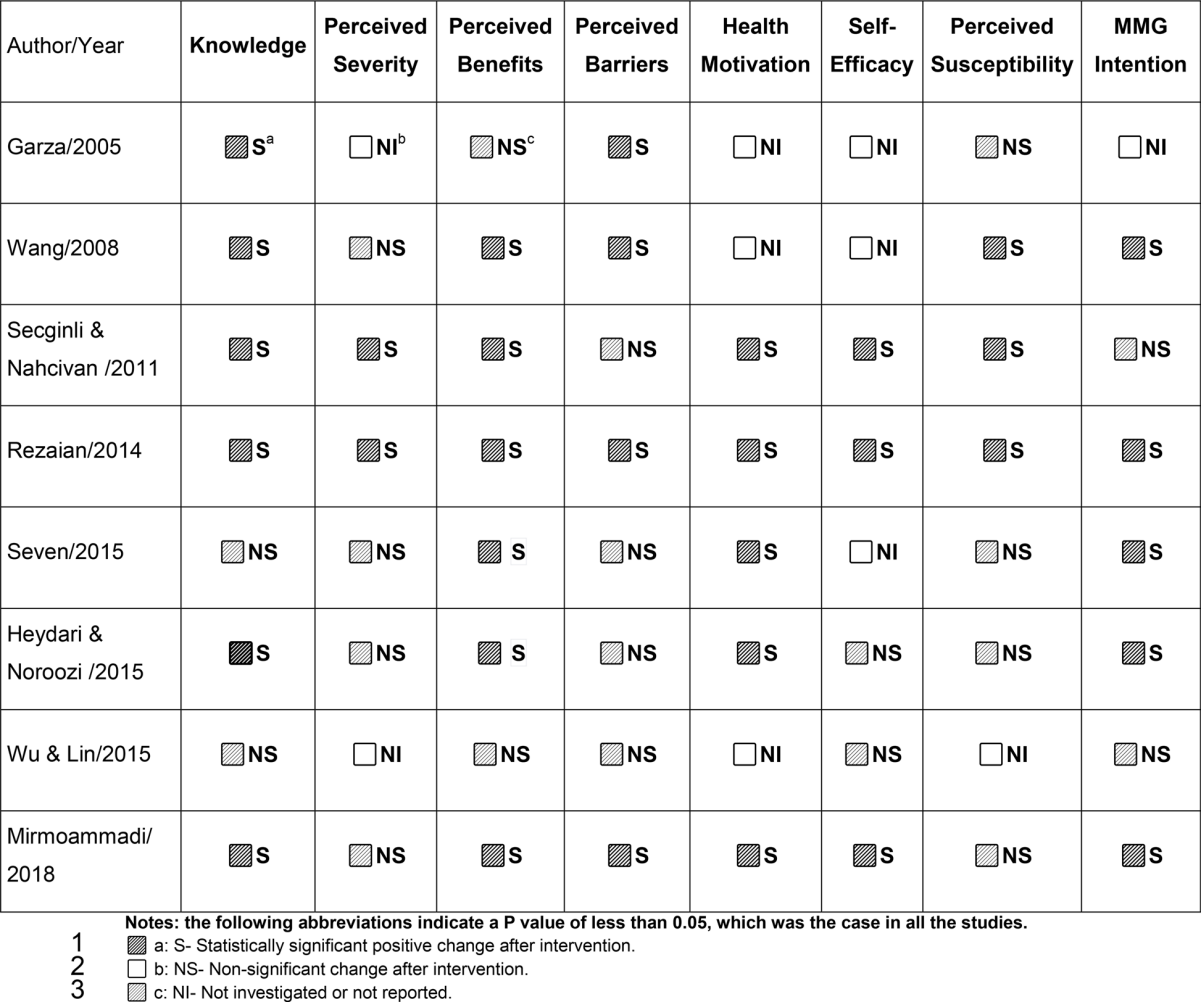
Summary of the studies’ knowledge, CHBMS, and intention outcomes

### Factors influencing screening intentions

Subgroup analysis revealed that certain socioeconomic and cultural factors moderated screening intentions. As reported in the included studies. Women with higher income and private health insurance were more likely to complete screenings. For example, Garza et al. (2005) reported that private insurance was a significant predictor of screening (*P* = 0.0486) [52, 54]. In contrast, low-income and minority women reported greater embarrassment, fear, perceived lack of necessity, and logistical constraints, which persisted after interventions [50, 54, 55]. Positive beliefs and knowledge retention also varied over time; Garza et al. (2005) observed a decrease in perceived barriers from 2.24 to 2.06 (*P* < 0.0001) [54], while Secginli & Nahcivan (2011) reported declines in knowledge at six-month follow-up [46]. While Mirmoammadi et al. (2018) showed significantly longer-term adherence through structured consultations [50]. Among the three studies focused on immigrant women in the U.S., one demonstrated that a culturally tailored video significantly improved knowledge (*P* < 0.001), various constructs of the CHBMS (*P* < 0.01), and the screening rate rose from 26.7% to 49.3% [55]. However, the other two studies had limited effects [52, 54].

Furthermore, several methodological limitations observed across the studies restrict the generalizability of the findings. These include small sample sizes [49, 50, 54, 55], reliance on convenience sampling [48, 49, 54, 55], and a lack of comparator groups in two studies [54, 55]. Additionally, self-reported outcomes may be affected by social desirability bias [46, 49, 50], and short follow-up periods [46, 50, 52, 54] limit the assessment of long-term intervention effects (Supplementary Table 8).

### HBM-based interventions outcomes

Six studies reported significant increases in BC knowledge scores after various interventions [46, 47, 49, 50, 54, 55]. For instance, Wang et al. (2008) found that the mean knowledge scores among Chinese American women increased from 7.36 to 8.43 (on a scale of 0–10; *P* = 0.001) after they viewed a culturally tailored educational video [55]. Similarly, Mirmoammadi et al. (2018) observed a significant increase in knowledge scores, from 45.09 to 73.75 (on a scale of 0–100; *P* < 0.001), following a three-month educational intervention [50]. However, Secginli and Nahcivan (2011) noted that the knowledge gains decreased six months after the intervention [46].

All studies included show improvement in at least one aspect of the CHBMS related to health beliefs, except one [52]. The constructs of perceived benefits and health motivation showed the most frequent improvement. For example, Heydari and Noroozi (2015) found that women receiving face-to-face group education scored significantly higher on perceived benefits (*P* = 0.029) and health motivation (*P* = 0.044) than those receiving multimedia education [49]. Furthermore, four studies reported reductions in perceived barriers, mainly when the interventions used interactive, face-to-face formats [48, 50, 54, 55].

Mammography intentions were generally improved in five studies following interventions [47–50, 55]. For instance, Rezaeian et al. (2014) found that women who perceive more benefits and fewer barriers to mammography are more likely to participate in screening [48]. Heydari and Noroozi (2015) found that, among 60 participants in each group, 56 (93%) from the education group and 50 (83%) from the multimedia group intended to undergo mammography. (*P* = 0.088). However, the translation of intention into behavior was limited [49]. In contrast, a study by Wu & Lin (2015) involving phone counseling found no significant difference in actual uptake between the intervention and control groups [52].

## Discussion

This SR assessed the effectiveness of HBM-based educational interventions to improve knowledge, beliefs, and intentions regarding mammogram screening among women aged 40 and older. Across the eight included studies (*n* = 1,439), interventions were generally effective in enhancing knowledge, beliefs, and intentions [46–50, 52, 54, 55]. These findings align with evidence from broader SRs and meta-analyses [25, 26, 29, 30] and with the foundational HBM literature [9, 23]. A SR by Agide et al. (2018) found that health promotion interventions leveraging behavioral models, such as the HBM, have successfully increased BCS participation over the past 12 years. This suggests that HBM-based interventions may serve as effective strategies for fostering screening practice [51]. Moreover, the predictive utility of the HBM in influencing screening behaviors has been widely validated in the literature [21, 28].

## Quality appraisal

The quality appraisal indicated that most of the RCTs (4 out of 5) had a low risk of bias, reinforcing the internal validity of their findings. These trials followed strict methodology and used validated outcome measures. In contrast, one RCT, while maintaining design integrity, lacked blinding, introducing potential performance and detection bias (51). Additionally, the assessment of three NRCTs showed a low risk of bias. Although the blinding of outcome assessors was poorly reported, structured recruitment processes and reliable measurement tools helped to reduce bias. However, the observational nature of NRCTs limits causal inference and generalizability. A key challenge in these studies was the difficulty of implementing blinding in educational interventions. Despite this, most studies demonstrated acceptable methodological rigor, aligning with recent SRs and meta-analyses that confirm the effectiveness of these interventions in increasing BCS rates, while acknowledging limitations in study design [18, 26, 32]. Methodological weaknesses, such as small sample sizes (43–327 participants), short follow-up periods (less than 6 months), and reliance on self-reported outcomes, were consistent with earlier reviews of screening interventions [16, 53].

### HBM-based interventions effectiveness

#### Knowledge effect

The observed improvements in knowledge following HBM-based interventions in most included studies are consistent with prior studies showing that theory-based education can improve BC awareness [10, 24, 30, 56]. For instance, Garza et al. (2005) and Wang et al. (2008) reported significant improvements in mammography-related knowledge and decreases in perceived barriers among minority women after culturally tailored interventions [54, 55]. These findings are consistent with a study conducted by Luque et al. (2018), who reported considerable improvements in mammography screening behaviors among Hispanic women when interventions were culturally adapted [24]. Likewise, a review by Huang Longcoy et al. (2023) demonstrated that educational interventions improved knowledge and mammography uptake among Asian American women, highlighting both the cultural specificity and delivery format of interventions as critical determinants of effectiveness [30]. These findings emphasize the need for culturally tailored education grounded in the HBM to improve knowledge and promote behavioral change, aligning with research on the importance of culturally and contextually relevant messaging [23, 34, 57, 58].

### Intervention characteristics effect

Notably, our review found that multifaceted interventions implemented within healthcare settings were more effective than unimodal or remote educational initiatives. This finding is consistent with the meta-analysis by Sohl and Moyer (2007), which highlighted the superiority of tailored, multi-component educational strategies over one-time or passive modalities [29]. Additionally, Matlabi et al. (2021) underscored the importance of volunteer-based, community-driven initiatives grounded in the HBM for promoting BCS in resource-constrained settings [27, 46]. Furthermore, this supports earlier research emphasizing that interactive educational formats foster greater engagement and stronger cognitive and emotional responses than passive information delivery [29, 51, 57].

### Sociocultural and systemic effect

Sociocultural, economic, and cultural determinants significantly moderated the effectiveness of interventions targeting mammography screening. Women with higher incomes, private health insurance, and greater access to healthcare services were more likely to complete their screenings [52, 54]. Interactive, face-to-face interventions demonstrated the most excellent efficacy in mitigating key psychosocial barriers, particularly embarrassment and fear [48, 54, 55]. Conversely, immigrant, low-income, and minority women encountered various barriers, including fatalistic beliefs and logistical challenges, which impeded their behavioral uptake despite advancements in knowledge [34, 59, 60]. These observations align with findings in global contexts, where cultural stigma and misinformation continue to be significant obstacles to breast health awareness and screening initiatives [17, 18].

### Health beliefs effect

The present review also demonstrates improvements across CHBM constructs, particularly highlighting perceived benefits and health motivation, in screening. Prior studies confirm that these are the strongest predictors of screening adherence, consistent with previous findings [20, 21, 28]. Furthermore, the persistence of perceived barriers such as cost, fear, and embarrassment highlights the challenges and the limitations of knowledge-based approaches in achieving sustained behavioral change [15, 18]. Conversely, our review indicates that perceived susceptibility and severity demonstrated less consistent changes. This trend corroborates the findings of Al-Sakkaf and Basaleem (2016) and Arevian et al. (2011), who observed that women frequently underestimate their susceptibility to BC while realizing its severity [6]. This suggests susceptibility-related beliefs may be rooted in fatalistic or cultural narratives that are harder to shift through one-off educational sessions [17, 18].

### Behavioral intention effect

The intention to undergo mammography increased across five studies [47–50, 55], indicating the potential of HBM-based interventions to enhance women’s motivation for screening, even in diverse cultural and socioeconomic populations. Notably, our review confirms that intention gains occur more reliably than actual behavioral uptake, a distinction also highlighted by Hagger and Weed (2019) in their debate on theory-driven health interventions [21, 28]. That is, while intentions reflect motivational readiness and are strongly predictive within theoretical models, they do not always translate into actual screening behavior due to persistent systemic, sociocultural, and logistical barriers [15, 18, 59]. Nduka et al. (2023) underscore this observation, noting that across lower-middle-income countries (LMICs), interventions frequently increase intentions. However, their impact on screening uptake remains inconsistent without reinforcement or health-system support [16]. It is important to note that this challenge is not unique to LMICs; high-income countries also struggle to bridge the intention-behavior gap, particularly among underserved populations, as seen in the Garza et al. (2005) study, where access to free mammography was a key factor in uptake [54, 60].

The decline in intention over time observed in some included studies (e.g., Heydari and Noroozi, 2015, where intention dropped from more than 90% to 80% at follow-up) aligns with broader evidence that one-off interventions have only a temporary influence [49]. Longitudinal findings from LMICs and community initiatives in sub-Saharan Africa reinforce the need for ongoing reinforcement strategies to sustain intentions and gradually facilitate behavior adoption [17, 31, 61].

### Considerations on the health belief model

This review discusses the HBM, noting its benefits and limitations. Primarily focused on Western, middle-class populations, the HBM may not fully represent the diverse health beliefs of various groups [60]. By emphasizing individual perceptions, it risks oversimplifying the complex cultural and social factors influencing women’s screening choices [62, 63]. (Bhargava et al., 2019; Ritchie et al., 2021) [64]. Additionally, its focus on belief modification may neglect important structural barriers, such as cost and access. Thus, while the HBM is a valuable educational tool, its effectiveness could be enhanced by considering broader contextual factors.

### Limitations and strengths

This SR has notable limitations that should be considered when interpreting the findings. First, it includes only eight studies, and the generally small sample sizes limit the generalizability of their individual findings. Second, the geographic scope was confined to the U.S., Iran, and Turkey, which further limits generalizability. Third, the variation in intervention methods and follow-up durations complicates conclusions about the sustainability of changes in knowledge, beliefs, and intentions. Fourth, reliance on self-reported outcomes raises the risk of recall and social desirability bias. Fifth, the exclusion of studies that utilized the HBM in combination with other theories or alternatives to the CHBMS may have led to the loss of relevant data.

Nevertheless, several strengths contribute to the validity of this SR. It employed a comprehensive search strategy across five databases, used standardized risk-of-bias assessment tools, and established clear eligibility criteria that prioritized interventional studies. This approach enhances causal inference and internal validity. Additionally, the narrative synthesis facilitated a broader investigation of intervention strategies. Ultimately, the review provides valuable insights into women’s knowledge, beliefs, and intentions regarding mammography, highlighting the effectiveness of HBM-based educational interventions in promoting screening services.

### Implications for practice and recommendations

Optimizing BCS interventions requires integrating additional health behavior theories alongside the HBM. Longitudinal studies are essential for assessing the sustainability of these interventions and promoting adherence across diverse sociocultural contexts. Healthcare providers should offer culturally tailored education as part of routine care, ensuring that their approaches align with each woman’s beliefs and address her specific barriers. Providing counselling, decision aids, and follow-up appointments can enhance women’s intentions to participate in screenings. Moreover, public health efforts should utilize interactive intervention methods and offer logistical support. Involving trusted community leaders and addressing cultural sensitivities can increase credibility among immigrant and underserved women. Additionally, technological solutions, such as mobile applications and telehealth, provide scalable opportunities for personalized education and support. Finally, systems and policies should incorporate BCS into national cancer control strategies, linking educational initiatives with improved access to care.

## Conclusion

The educational interventions based on the HBM, as examined in the eight studies included in this SR, were generally effective in enhancing BC knowledge, fostering positive health beliefs, and improving intentions regarding mammography among women aged 40 and older. However, these improvements do not consistently lead to increased screening rates due to logistical constraints and socioeconomic and cultural factors that moderate these effects. While the overall risk of bias is low, limitations such as small sample sizes, reliance on self-reporting, short follow-up periods, and diverse intervention delivery methods and outcomes hinder the ability to conduct a meta-analysis. To effectively translate improved beliefs into behavioral change, future initiatives should adopt a culturally tailored, multifaceted approach integrated within healthcare systems. Rigorous studies with adequate sample sizes, diverse populations, and extended follow-up periods are essential to enhance adherence to screening guidelines and reduce BC mortality.

## Supplementary Information

Supplementary Material 1.

Supplementary Material 2.

Supplementary Material 3.

Supplementary Material 4.

Supplementary Material 5.

Supplementary Material 6.

Supplementary Material 7.

Supplementary Material 8.

Supplementary Material 9.

Supplementary Material 10.

Supplementary Material 11.

## Data Availability

All data generated or analyzed during this study are included in this published article [and its supplementary files].

## Abbreviations

ACR: American College of Radiology
BC: Breast Cancer
BCS: Breast Cancer Screening
BSE: Breast Self-Examination
CBE: Clinical Breast Examination
CHBM: Champions’ Health Belief Model
CHBMS: Champions’ Health Belief Model Scale
HBM: Health Belief Model
IARC: International Agency for Research on Cancer
LMICs: Lower-middle-income countries
NCCN: National Comprehensive Cancer Network
NRCT: Nonrandomized Controlled Trials
MeSH: Medical Subject Headings
PICOS: Population, Intervention, Comparator, Outcome, and Study design
PRISMA: Preferred Reporting Items for Systematic Review and Meta-Analysis
PROSPERO: Prospective Register of Systematic Reviews
RCT: Randomized Controlled Trials
Rob 2: Cochrane Risk of Bias 2 tool
RoBANS 2: Revised Risk of Bias Assessment 2 tool
SCT: Social Cognitive Theory
SR: Systematic Review
TPB: Theory of Planned Behavior
U.S.: United States
