# Supplementary figures and images for: Health belief model-based educational interventions for knowledge, beliefs, and intentions on mammography: a systematic review
Source: BMC Womens Health. 2025 Dec 22;26:48. doi: 10.1186/s12905-025-04218-9 (PMC12836963; doi:10.1186/s12905-025-04218-9)

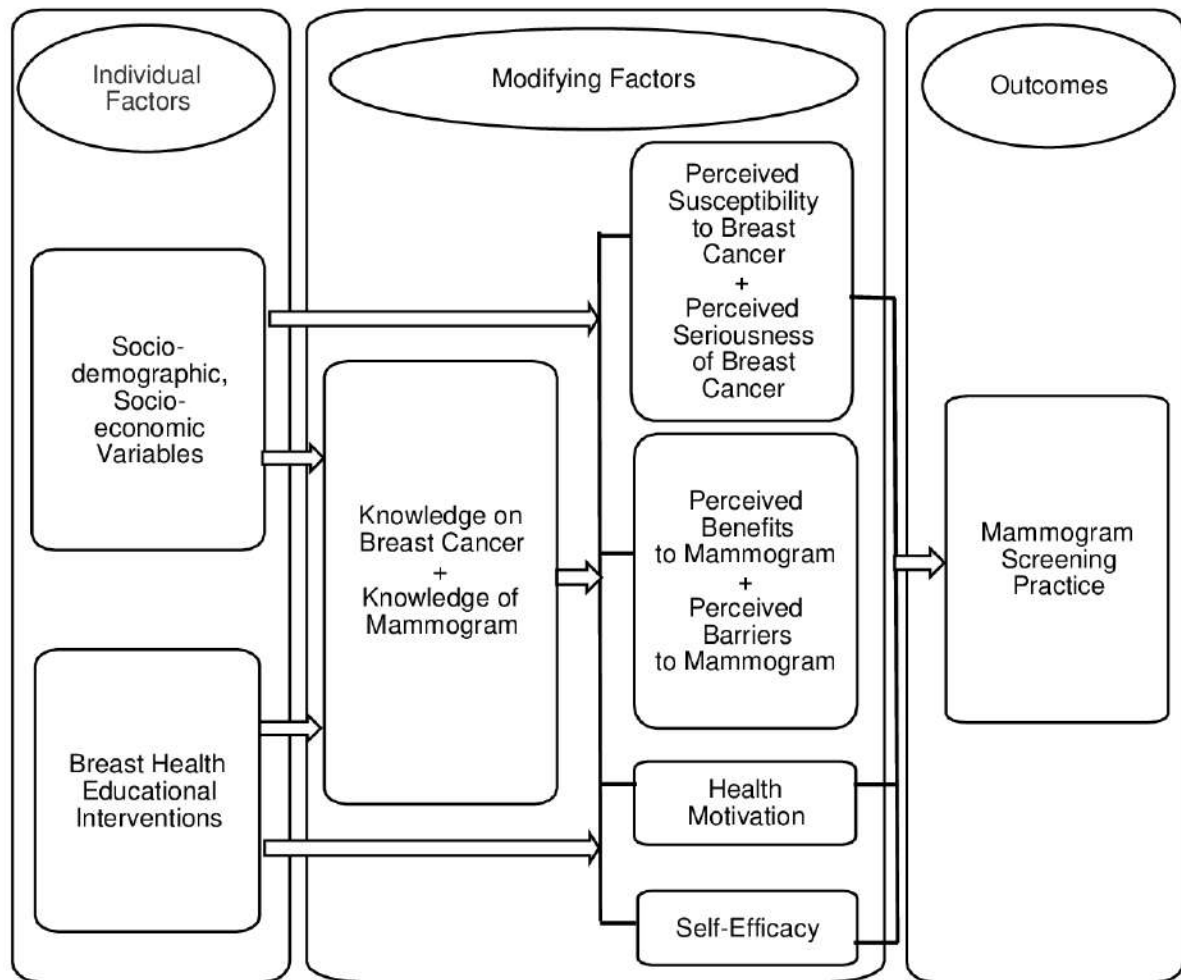

Supplement: Supplementary file 3 — Supplementary Material 3. [file 12905_2025_4218_MOESM3_ESM.pdf]
