# Supplementary material for: Health belief model-based educational interventions for knowledge, beliefs, and intentions on mammography: a systematic review
Source: BMC Womens Health. 2025 Dec 22;26:48. doi: 10.1186/s12905-025-04218-9 (PMC12836963; doi:10.1186/s12905-025-04218-9)
Supplement: Supplementary file 4 — Supplementary Material 4. [file 12905_2025_4218_MOESM4_ESM.docx]

**Supplementary Table 1: Documentation Template for the Search Strategy**

**Database 1:** PubMed

| # | Search String | Results  2023 | Results  2024 |
| --- | --- | --- | --- |
| 1 | "Breast Neoplasms"[Mesh] OR "Breast Neoplasm*"[tiab] OR "Breast Tumor*"[tiab] OR "Tumor,  Breast"[tiab] OR "Tumors, Breast"[tiab] OR "Neoplasm*, Breast"[tiab] OR "Breast Cancer" [tiab] OR "Cancer, Breast"[tiab] OR "Mammary Cancer"[tiab] OR "Cancer, Mammary" [tiab] OR "Cancers, Mammary"[tiab] OR "Mammary Cancers"[tiab] OR Malignant Neoplasm of  Breast" [tiab] OR "Breast Malignant Neoplasm"[tiab] OR "Breast Malignant Neoplasms" [tiab] OR "Malignant Tumor of Breast"[tiab] OR "Breast Malignant Tumor"[tiab] OR "Breast Malignant Tumors"[tiab] OR "Cancer of Breast"[tiab] OR "Cancer of the Breast"[tiab] OR "Breast Carcinoma"[tiab] OR "Breast Carcinomas"[tiab] OR "Carcinoma, Breast"[tiab] OR "Carcinomas,  Breast"[tiab] OR "Mammary Glands"[tiab] OR "Human Mammary Glands"[tiab] | 449.762 | 490,683 |
| 2 | "Health Belief Model"[Mesh] OR "Health Belief Model"[tiab] OR "Champion's Health Belief Model"[tiab] OR "Champion's Health Belief Model Scale"[tiab] OR "Revised Champion’s Health Belief Model Scale"[tiab] | 3,651 | 4,311 |
| 3 | Mammography[Mesh] OR "Mass Screening"[Mesh] OR "Early Detection of Cancer"[Mesh]  OR Diagnosis[Mesh] OR "screening mammography"[tiab] OR "screening mammogram*" [tiab] OR  "screening practices"[tiab] OR "screening behavior"[tiab] OR "screening test” [tiab] OR "breast cancer screening"[tiab] OR "breast cancer detection"[tiab] OR “breast cancer diagnosis"[tiab] OR "early breast cancer detection"[tiab] OR "early breast cancer screening" [tiab] OR "early breast cancer diagnosis"[tiab] OR "early detection" [tiab] OR "early diagnosis" [tiab] OR "early screening"[tiab] OR Mammography[tiab] OR Mammogram [tiab] OR Screening[tiab] OR Practice[tiab] OR diagnosis[tiab] | 11,177,414 | [11,848,479](https://pubmed.ncbi.nlm.nih.gov/?term=Mammography%5BMesh%5D+OR+%22Mass+Screening%22%5BMesh%5D+OR+%22Early+Detection+of+Cancer%22%5BMesh%5D+OR+Diagnosis%5BMesh%5D+OR+%22screening+mammography%22%5Btiab%5D+OR+%22screening+mammogram%2A%22%5Btiab%5D+OR+%22screening+practices%22%5Btiab%5D+OR+%22screening+behavior%22%5Btiab%5D+OR+%22screening+tests%22%5Btiab%5D+OR+%22breast+cancer+screening%22%5Btiab%5D+OR+%22breast+cancer+detection%22%5Btiab%5D+OR+%22breast+cancer+diagnosis%22%5Btiab%5D+OR+%22early+breast+cancer+detection%22%5Btiab%5D+OR+%22early+breast+cancer+screening%22%5Btiab%5D+OR+%22early+breast+cancer+diagnosis%22%5Btiab%5D+OR+%22early+detection%22+%5Btiab%5D+OR+%22early+diagnosis%22%5Btiab%5D+OR+%22early+screening%22%5Btiab%5D+OR+Mammography%5Btiab%5D+OR+Mammogram%5Btiab%5D+OR+Screening%5Btiab%5D+OR+Practice%5Btiab%5D++OR+diagnosis%5Btiab%5D&sort=) |
| 4 | Education [Mesh] OR Education[tiab] OR "education activiti*"[tiab] OR "education intervention*"[tiab] OR "education program*"[tiab] | 1,278,273 | [1,384,593](https://pubmed.ncbi.nlm.nih.gov/?term=Education+%5BMesh%5D+OR+Education%5Btiab%5D+OR+%22education+activiti%2A%22%5Btiab%5D+OR+%22education+intervention%2A%22%5Btiab%5D+OR+%22education+program%2A%22%5Btiab%5D&sort=) |
| 5 | "Health Knowledge, Attitudes, Practice"[Mesh] OR "Health Behavior"[tiab] OR "Health Knowledge, Attitudes, Practice"[tiab] OR "health beliefs"[tiab] OR knowledge[tiab] OR  culture[Mesh] OR practice[tiab] OR behavior[tiab] OR attitude[tiab] OR belief[tiab] OR Awareness[Mesh] OR awareness[tiab] OR culture[tiab] | 3,416,108 | [3,787,934](https://pubmed.ncbi.nlm.nih.gov/?term=%22Health+Knowledge%2C+Attitudes%2C+Practice%22%5BMesh%5D+OR+%22Health+Behavior%22%5Btiab%5D+OR+%22Health+Knowledge%2C+Attitudes%2C+Practice%22%5Btiab%5D+OR+%22health+beliefs%22%5Btiab%5D+OR+knowledge%5Btiab%5D+OR+culture%5BMesh%5D+OR+practice%5Btiab%5D+OR+behavior%5Btiab%5D+OR+attitude%5Btiab%5D+OR+belief%5Btiab%5D+OR+Awareness%5BMesh%5D+OR+awareness%5Btiab%5D+OR+culture%5Btiab%5D&sort=) |
| 6 | Women[Mesh] OR Female[Mesh] OR Women[tiab] OR Female[tiab] | 9,886,810 | [10,393,683](https://pubmed.ncbi.nlm.nih.gov/?term=Women%5BMesh%5D+OR+Female%5BMesh%5D+OR+Women%5Btiab%5D+OR+Female%5Btiab%5D&sort=) |
| 7 | #1 AND #2 AND #3 AND #4 AND #5 AND #6 | 147 | 157 |
| 8 | #1 AND #2 AND #3 AND #4 AND #5 AND #6 **Filters: from 2003 – 2023** | 118 |  |
|  | #1 AND #2 AND #3 AND #4 AND #5 AND #6 **Filters: from 2003 – 2024** |  | 129 |
| 9 | #1 AND #2 AND #3 AND #4 AND #5 AND #6 **Filters: English, from 2003 - 2023** | 117 |  |
|  | #1 AND #2 AND #3 AND #4 AND #5 AND #6 **Filters: English, from 2003 - 2024** |  | 128 |

**Database 2:** EMBASE

| # | Search String | Results  2023 | Results  2024 |
| --- | --- | --- | --- |
| 1 | exp "Breast Neoplasms"/ OR "Breast Neoplasm*".tw. OR "Breast Tumor*".tw. OR "Tumor, Breast".tw. OR "Tumors, Breast".tw. OR "Neoplasm*, Breast".tw. OR "Breast Cancer".tw.  OR "Cancer, Breast".tw. OR "Mammary Cancer".tw. OR "Cancer, Mammary".tw. OR  "Cancers, Mammary".tw. OR "Mammary Cancers".tw. OR "Malignant Neoplasm of Breast".tw. OR "Breast Malignant Neoplasm".tw. OR "Breast Malignant Neoplasms".  tw. OR "Malignant Tumor of Breast".tw. OR "Breast Malignant Tumor".tw. OR "Breast Malignant Tumors".tw. OR "Cancer of Breast".tw. OR "Cancer of the Breast".tw. OR  "Breast Carcinoma".tw. OR "Breast Carcinomas".tw. OR "Carcinoma, Breast".tw. OR  "Carcinomas, Breast".tw. OR "Mammary Glands".tw. OR "Human Mammary Glands".tw. | 409,782 | 505,101 |
| 2 | exp "Health Belief Model"/ OR "Health Belief Model".tw. OR "Champion's Health Belief Model".tw. OR "Champion's Health Belief Model Scale".tw. OR "Revised Champion’s Health Belief Model Scale".tw. | 4,227 | 5,206 |
| 3 | exp Mammography/ OR exp "Mass Screening"/ OR exp "Early Detection of Cancer"/ OR  exp Diagnosis/ OR "screening mammography".tw. OR "screening mammogram*".tw.  OR "screening practices".tw. OR "screening behavior".tw. OR "screening tests".tw. OR  "breast cancer screening".tw. OR "breast cancer detection".tw. OR "breast cancer diagnosis".tw. OR "early breast cancer detection".tw. OR "early breast cancer screening"  .tw. OR "early breast cancer diagnosis".tw. OR "early detection".tw. OR "early diagnosis” .tw. OR "early screening".tw. OR Mammography.tw. OR Mammogram.tw. OR Screening  .tw. OR Practice.tw. OR diagnosis.tw. | 10,183,085 | 12,654,921 |
| 4 | exp Education/ OR Education.tw. OR "education activiti*".tw. OR "education intervention*".tw. OR "education program*".tw. | 1,873,368 | 3,104,797 |
| 5 | exp "Health Knowledge, Attitudes, Practice"/ OR "Health Behavior".tw. OR "Health Knowledge, Attitudes, Practice".tw. OR "health beliefs".tw. OR knowledge.tw. OR exp  culture/ OR practice.tw. OR behavior.tw. OR attitude.tw. OR belief.tw. OR exp Awareness/ OR awareness.tw. OR culture.tw. | 4,107,070 | 5,277,167 |
| 6 | exp Women/ OR exp Female/ OR Women.tw. OR Female.tw. | 11,702,956 | 11,047,752 |
| 7 | 1 and 2 and 3 and 4 and 5 and 6 | 174 | 231 |
| 8 | limit 7 to (English language and yr="2003 - 2023") | 147 |  |
|  | limit 7 to (English language and yr="2003 - 2024") |  | 177 |

**Database 3:** CINAHL

| # | Search string | Results  2023 | Results  2024 |
| --- | --- | --- | --- |
| 1 | (MH "Breast Neoplasms+") OR (TI " Breast Neoplasm*" OR AB " Breast Neoplasm*") OR  (TI "Breast Tumor*" OR AB "Breast Tumor*") OR (TI "Tumor, Breast" OR AB "Tumor, Breast") OR (TI "Tumors, Breast" OR AB "Tumors, Breast") OR (TI "Neoplasm*, Breast"   OR AB "Neoplasm*, Breast") OR (TI "Breast Cancer" OR AB "Breast Cancer") OR (T1  "Cancer, Breast" OR AB "Cancer, Breast") OR (TI "Mammary Cancer" OR AB “Mammary  Cancer") OR (TI "Cancer, Mammary" OR AB "Cancer, Mammary") OR (TI "Cancers, Mammary" OR AB "Cancers, Mammary") OR (TI "Mammary Cancers" OR AB "Mammary Cancers") OR (TI "Malignant Neoplasm of Breast" OR AB "Malignant Neoplasm of Breast") OR (TI "Breast Malignant Neoplasm" OR AB "Breast Malignant Neoplasm") OR  (TI "Breast Malignant Neoplasms" OR AB "Breast Malignant Neoplasms") OR (TI  "Malignant Tumor of Breast" OR AB "Malignant Tumor of Breast") OR (TI "Breast Malignant Tumor" OR AB "Breast Malignant Tumor") OR (TI "Breast Malignant Tumors"  OR AB "Breast Malignant Tumors") OR (TI "Cancer of Breast" OR AB "Cancer of Breast”) OR (TI "Cancer of the Breast" OR AB "Cancer of the Breast") OR (TI "Breast Carcinoma"   OR AB "Breast Carcinoma") OR (TI "Breast Carcinomas" OR AB "Breast Carcinomas")  OR (TI "Carcinoma, Breast" OR AB "Carcinoma, Breast") OR (TI "Carcinomas, Breast" OR  AB "Carcinomas, Breast") OR (TI "Mammary Glands" OR AB "Mammary Glands") OR (TI  "Human Mammary Glands" OR AB "Human Mammary Glands") | 117,974 | 125,303 |
| 2 | (MH "Health Belief Model+") OR (TI "Health Belief Model" OR AB "Health Belief Model")  OR (TI "Champion's Health Belief Model" OR AB "Champion's Health Belief Model") OR   (TI "Champion's Health Belief Model Scale" OR AB "Champion's Health Belief Model Scale") OR (TI "Revised Champion’s Health Belief Model Scale" OR AB "Revised Champion’s Health Belief Model Scale") | 3,585 | 3,849 |
| 3 | ((MH Mammography+) OR (MH "Mass Screening+") OR (MH "Early Detection of Cancer+") OR (MH Diagnosis+) OR (TI "screening mammography" OR AB "screening mammography") OR (TI "screening mammogram*." OR AB "screening mammogram*.) OR (TI "screening practices" OR AB "screening practices") OR (TI "screening behavior" OR  AB "screening behavior") OR (TI "screening tests" OR AB "screening tests") OR (TI "breast  cancer screening" OR AB "breast cancer screening") OR (TI "breast cancer detection” OR AB "breast cancer detection") OR (TI "breast cancer diagnosis" OR AB "breast cancer diagnosis") OR (TI "early breast cancer detection" OR AB "early breast cancer detection")  OR (TI "early breast cancer screening" OR AB "early breast cancer screening") OR (TI  "early breast cancer diagnosis" OR AB "early breast cancer diagnosis") OR (TI "early detection" OR AB "early detection") OR (TI "early diagnosis" OR AB "early diagnosis")  OR (TI "early screening" OR AB "early screening") OR (TI Mammography OR AB Mammography) OR (TI Mammogram OR AB Mammogram) OR (TI Screening OR AB Screening) OR (TI Practice OR AB Practice) OR (TI diagnosis OR AB diagnosis) | 2,820,235 | 2,924,121 |
| 4 | (MH Education+) OR (TI Education OR AB Education) OR (TI "education activiti*" OR AB  "education activiti*") OR (TI "education intervention*" OR AB "education intervention*") OR  (TI "education program*" OR AB "education program*") | 1,167,461 | 1,235,154 |
| 5 | (MH "Health Knowledge, Attitudes, Practice+") OR (TI "Health Behavior" OR AB "Health Behavior") OR (TI "Health Knowledge, Attitudes, Practice" OR AB "Health Knowledge, Attitudes, Practice") OR (TI "health beliefs" OR AB "health beliefs") OR (TI knowledge OR  AB knowledge) OR (MH culture+) OR (TI practice OR AB practice) OR (TI behavior OR AB behavior) OR (TI attitude OR AB attitude) OR (TI belief OR AB belief) OR (MH Awareness+) OR (TI awareness OR AB awareness) OR (TI culture OR AB culture) | 1,302,586 | 1,350,641 |
| 6 | (MH Women+) OR (MH Female+) OR (TI Women OR AB Women) OR (TI Female OR AB Female) | 2,367,861 | 2,458,644 |
| 7 | S1 AND S2 AND S3 AND S4 AND S5 AND S6 | 182 | 182 |
| 8 | S1 AND S2 AND S3 AND S4 AND S5 AND S6 -- Limiters - Published Date: 2003-2023 | 128 |  |
|  | S1 AND S2 AND S3 AND S4 AND S5 AND S6 -- Limiters - Published Date: 2003-2024 |  | 129 |
| 9 | S1 AND S2 AND S3 AND S4 AND S5 AND S6---Limiters (Published Date: 2003-2023, English language) | 120 |  |
|  | S1 AND S2 AND S3 AND S4 AND S5 AND S6---Limiters (Published Date: 2003-2024, English language) |  | 122 |

**Database 4:** Web of Science

| # | Search string | Results  2023 | Results  2024 |
| --- | --- | --- | --- |
| 1 | (TI="Breast Neoplasm*" OR AB="Breast Neoplasm*") OR (TI="Breast Tumor*" OR AB  ="Breast Tumor*") OR (TI="Tumor, Breast" OR AB="Tumor, Breast") OR (TI="Tumors, Breast" OR AB="Tumors, Breast") OR (TI="Neoplasm*, Breast" OR AB="Neoplasm*, Breast") OR (TI="Breast Cancer" OR AB="Breast Cancer") OR (TI="Cancer, Breast" OR  AB="Cancer, Breast") OR (TI="Mammary Cancer" OR AB="Mammary Cancer") OR (TI=  "Cancer, Mammary" OR AB="Cancer, Mammary") OR (TI="Cancers, Mammary" OR AB  ="Cancers, Mammary") OR (TI="Mammary Cancers" OR AB="Mammary Cancers") OR  (TI="Malignant Neoplasm of Breast" OR AB="Malignant Neoplasm of Breast") OR (TI  ="Breast Malignant Neoplasm" OR AB="Breast Malignant Neoplasm") OR (TI="Breast Malignant Neoplasms" OR AB="Breast Malignant Neoplasms") OR (TI="Malignant Tumor of Breast" OR AB="Malignant Tumor of Breast") OR (TI="Breast Malignant Tumor" OR AB  ="Breast Malignant Tumor") OR (TI="Breast Malignant Tumors" OR AB="Breast Malignant Tumors") OR (TI="Cancer of Breast" OR AB="Cancer of Breast") OR (TI="Cancer of the Breast" OR AB="Cancer of the Breast") OR (TI="Breast Carcinoma" OR AB="Breast Carcinoma") OR (TI="Breast Carcinomas" OR AB="Breast Carcinomas") OR (TI=  "Carcinoma, Breast" OR AB="Carcinoma, Breast") OR (TI="Carcinomas, Breast" OR AB  ="Carcinomas, Breast") OR (TI="Mammary Glands" OR AB="Mammary Glands") OR (TI  ="Human Mammary Glands" OR AB="Human Mammary Glands") | 424,095 | 515,063 |
| 2 | (TI="Health Belief Model" OR AB="Health Belief Model") OR (TI="Champion's Health Belief Model" OR AB="Champion's Health Belief Model") OR (TI="Champion's Health Belief Model Scale" OR AB="Champion's Health Belief Model Scale") OR (TI="Revised Champion’s Health Belief Model Scale" OR AB="Revised Champion’s Health Belief Model Scale") | 3,200 | 4,302 |
| 3 | (TI=Mammography OR AB=Mammography) OR (TI="Mass Screening" OR AB="Mass Screening") OR (TI="Early Detection of Cancer" OR AB="Early Detection of Cancer") OR  (TI=Diagnosis OR AB=Diagnosis) OR (TI="screening mammography" OR AB="screening mammography") OR (TI="screening mammogram*." OR AB="screening mammogram*.")  OR (TI="screening practices" OR AB="screening practices") OR (TI="screening behavior" OR AB="screening behavior") OR (TI="screening tests" OR AB="screening tests") OR (TI=  "breast cancer screening" OR AB="breast cancer screening") OR (TI= "breast cancer detection" OR AB="breast cancer detection") OR (TI="breast cancer diagnosis" OR AB=  "breast cancer diagnosis") OR (TI="early breast cancer detection" OR AB="early breast cancer detection") OR (TI="early breast cancer screening" OR AB="early breast cancer screening") OR (TI="early breast cancer diagnosis" OR AB="early breast cancer diagnosis” ) OR (TI="early detection" OR AB="early detection") OR (TI="early diagnosis" OR AB=  "early diagnosis") OR (TI="early screening" OR AB="early screening")  OR (TI=  Mammogram OR AB=Mammogram) OR (TI=Screening OR AB=Screening) OR (TI=  Practice OR AB=Practice) | 4,284,646 | 5,617,942 |
| 4 | (TI=Education OR AB=Education) OR (TI="education activiti*" OR AB="education activiti*")  OR (TI="education intervention*" OR AB="education intervention*") OR (TI="education  program*" OR AB="education program*") | 954,267 | 1,415,751 |
| 5 | (TI="Health Behavior" OR AB="Health Behavior") OR (TI="Health Knowledge, Attitudes, Practice" OR AB="Health Knowledge, Attitudes, Practice") OR (TI="health beliefs" OR AB=  "health beliefs") OR (TI=knowledge OR AB=knowledge) OR (TI=practice OR AB=practice)  OR (TI=behavior OR AB=behavior) OR (TI=attitude OR AB=attitude) OR (TI=belief OR AB=belief) OR (TI=awareness OR AB=awareness) OR (TI=culture OR AB=culture) | 8,072,797 | 10,426,666 |
| 6 | (TI=Women OR AB=Women) OR (TI=Female OR AB=Female) | 2,461,619 | 3,212,846 |
| 7 | #6 AND #5 AND #4 AND #3 AND #2 AND #1 | 66 | 80 |
| 8 | #6 AND #5 AND #4 AND #3 AND #2 AND #1 and 2005 or 2006 or 2007 or 2008 or 2009 or 2010 or 2011 or 2012 or 2013 or 2014 or 2015 or 2016 or 2017 or 2018 or 2019 or 2020 or 2021 or 2022 or 2023 (Publication) | 56 |  |
|  | #6 AND #5 AND #4 AND #3 AND #2 AND #1 and 2005 or 2006 or 2007 or 2008 or 2009 or 2010 or 2011 or 2012 or 2013 or 2014 or 2015 or 2016 or 2017 or 2018 or 2019 or 2020 or 2021 or 2022 or 2023 or 2024 (Publication) |  | 70 |
| 9 | #6 AND #5 AND #4 AND #3 AND #2 AND #1 and 2005 or 2006 or 2007 or 2008 or 2009 or 2010 or 2011 or 2012 or 2013 or 2014 or 2015 or 2016 or 2017 or 2018 or 2019 or 2020 or 2021 or 2022 or 2023 (Publication Years) and English (Languages) | 56 |  |
|  | #6 AND #5 AND #4 AND #3 AND #2 AND #1 and 2005 or 2006 or 2007 or 2008 or 2009 or 2010 or 2011 or 2012 or 2013 or 2014 or 2015 or 2016 or 2017 or 2018 or 2019 or 2020 or 2021 or 2022 or 2023 or 2024 (Publication Years) and English (Languages) |  | 69 |

**Database 5:** Web of Science

| # | Search string | Results  2023 | Results  2024 |
| --- | --- | --- | --- |
| 1 | (TI="Breast Neoplasm*" OR AB="Breast Neoplasm*") OR (TI="Breast Tumor*" OR AB=  "Breast Tumor*") OR (TI="Tumor, Breast" OR AB="Tumor, Breast") OR (TI="Tumors, Breast" OR AB="Tumors, Breast") OR (TI="Neoplasm*, Breast" OR AB="Neoplasm*, Breast") OR (TI="Breast Cancer" OR AB="Breast Cancer") OR (TI="Cancer, Breast" OR  AB="Cancer, Breast") OR (TI="Mammary Cancer" OR AB="Mammary Cancer") OR (TI=  "Cancer, Mammary" OR AB="Cancer, Mammary") OR (TI="Cancers, Mammary" OR AB=  "Cancers, Mammary") OR (TI="Mammary Cancers" OR AB="Mammary Cancers") OR  (TI="Malignant Neoplasm of Breast" OR AB="Malignant Neoplasm of Breast") OR (TI=  "Breast Malignant Neoplasm" OR AB="Breast Malignant Neoplasm") OR (TI="Breast Malignant Neoplasms" OR AB="Breast Malignant Neoplasms") OR (TI="Malignant Tumor of Breast" OR AB="Malignant Tumor of Breast") OR (TI="Breast Malignant Tumor" OR   AB="Breast Malignant Tumor") OR (TI="Breast Malignant Tumors" OR AB="Breast Malignant Tumors") OR (TI="Cancer of Breast" OR AB="Cancer of Breast") OR (TI=  "Cancer of the Breast" OR AB="Cancer of the Breast") OR (TI="Breast Carcinoma" OR  AB="Breast Carcinoma") OR (TI="Breast Carcinomas" OR AB="Breast Carcinomas") OR  (TI="Carcinoma, Breast" OR AB="Carcinoma, Breast") OR (TI="Carcinomas, Breast" OR   AB="Carcinomas, Breast") OR (TI="Mammary Glands" OR AB="Mammary Glands") OR   (TI="Human Mammary Glands" OR AB="Human Mammary Glands") | 424,095 | 515,063 |
| 2 | (TI="Health Belief Model" OR AB="Health Belief Model") OR (TI="Champion's Health Belief Model" OR AB="Champion's Health Belief Model") OR (TI="Champion's Health Belief Model Scale" OR AB="Champion's Health Belief Model Scale") OR (TI="Revised Champion’s Health Belief Model Scale" OR AB="Revised Champion’s Health Belief Model Scale") | 3,200 | 4,302 |
| 3 | (TI=Mammography OR AB=Mammography) OR (TI="Mass Screening" OR AB="Mass Screening") OR (TI="Early Detection of Cancer" OR AB="Early Detection of Cancer")  OR (TI=Diagnosis OR AB=Diagnosis) OR (TI="screening mammography" OR AB=  "screening mammography") OR (TI="screening mammogram*." OR AB="screening mammogram*.") OR (TI="screening practices" OR AB="screening practices") OR (TI=  "screening behavior" OR AB="screening behavior") OR (TI="screening tests" OR AB=  "screening tests") OR (TI="breast cancer screening" OR AB="breast cancer screening")  OR (TI="breast cancer detection" OR AB="breast cancer detection") OR (TI="breast  cancer diagnosis" OR AB="breast cancer diagnosis") OR (TI="early breast cancer detection" OR AB="early breast cancer detection") OR (TI="early breast cancer screening" OR AB="early breast cancer screening") OR (TI="early breast cancer diagnosis" OR AB="early breast cancer diagnosis") OR (TI="early detection" OR AB=  "early detection") OR (TI="early diagnosis" OR AB="early diagnosis") OR (TI="early screening" OR AB="early screening")  OR (TI=Mammogram OR AB=Mammogram) OR  (TI=Screening OR AB=Screening) OR (TI=Practice OR AB=Practice) | 4,284,646 | 5,617,942 |
| 4 | (TI=Education OR AB=Education) OR (TI="education activiti*" OR AB="education activiti*") OR (TI="education intervention*" OR AB="education intervention*") OR  (TI="education program*" OR AB="education program*") | 954,267 | 1,415,751 |
| 5 | (TI="Health Behavior" OR AB="Health Behavior") OR (TI="Health Knowledge, Attitudes, Practice" OR AB="Health Knowledge, Attitudes, Practice") OR (TI="health beliefs" OR  AB="health beliefs") OR (TI=knowledge OR AB=knowledge) OR (TI=practice OR AB=  practice) OR (TI=behavior OR AB=behavior) OR (TI=attitude OR AB=attitude) OR (TI=belief OR AB=belief) OR (TI=awareness OR AB=awareness) OR (TI=culture OR AB=culture) | 8,072,797 | 10,426,666 |
| 6 | (TI=Women OR AB=Women) OR (TI=Female OR AB=Female) | 2,461,619 | 3,212,846 |
| 7 | #6 AND #5 AND #4 AND #3 AND #2 AND #1 | 66 | 80 |
| 8 | #6 AND #5 AND #4 AND #3 AND #2 AND #1 and 2005 or 2006 or 2007 or 2008 or 2009 or 2010 or 2011 or 2012 or 2013 or 2014 or 2015 or 2016 or 2017 or 2018 or 2019 or 2020 or 2021 or 2022 or 2023 (Publication) | 56 |  |
|  | #6 AND #5 AND #4 AND #3 AND #2 AND #1 and 2005 or 2006 or 2007 or 2008 or 2009 or 2010 or 2011 or 2012 or 2013 or 2014 or 2015 or 2016 or 2017 or 2018 or 2019 or 2020 or 2021 or 2022 or 2023 or 2024 (Publication) |  | 70 |
| 9 | #6 AND #5 AND #4 AND #3 AND #2 AND #1 and 2005 or 2006 or 2007 or 2008 or 2009 or 2010 or 2011 or 2012 or 2013 or 2014 or 2015 or 2016 or 2017 or 2018 or 2019 or 2020 or 2021 or 2022 or 2023 (Publication Years) and English (Languages) | 56 |  |
|  | #6 AND #5 AND #4 AND #3 AND #2 AND #1 and 2005 or 2006 or 2007 or 2008 or 2009 or 2010 or 2011 or 2012 or 2013 or 2014 or 2015 or 2016 or 2017 or 2018 or 2019 or 2020 or 2021 or 2022 or 2023 or 2024 (Publication Years) and English (Languages) |  | 69 |
