# Supplementary material for: Health belief model-based educational interventions for knowledge, beliefs, and intentions on mammography: a systematic review
Source: BMC Womens Health. 2025 Dec 22;26:48. doi: 10.1186/s12905-025-04218-9 (PMC12836963; doi:10.1186/s12905-025-04218-9)
Supplement: Supplementary file 5 — Supplementary Material 5. [file 12905_2025_4218_MOESM5_ESM.docx]

**Supplementary Table 2: Summary of Search Across Five Electronic Databases**

| Name of Database | | Platform | | | Date Range | Searched Date | | | No. of Results | | | |
| --- | --- | --- | --- | --- | --- | --- | --- | --- | --- | --- | --- | --- |
| PubMed | | PubMed | | | 2003 – 2023 | 09/04/2023 | | | 117 | | | |
|  |  |  |  |  | 2003 – 2024 | 07/12/2024 | | | 127 | | | |
| EMBASE | | Ovid | | | 2003 – 2023 | 09/04/2023 | | | 147 | | | |
|  |  |  |  |  | 2003 – 2024 | 07/12/2024 | | | 177 | | | |
| CINAHL | | EBSCO | | | 2003 – 2023 | 09/04/2023 | | | 120 | | | |
|  |  |  |  |  | 2003 – 2024 | 07/12/2024 | | | 122 | | | |
| Web of Science | | Web of Science | | | 2003 – 2023 | 09/04/2023 | | | 56 | | | |
|  |  |  |  |  | 2003 – 2024 | 07/12/2024 | | | 69 | | | |
| PsycINFO | | EBSCO | | | 2003 – 2023 | 09/04/2023 | | | 28 | | | |
|  |  |  |  |  | 2003 – 2024 | 07/12/2024 | | | 62 | | | |
| SR Screening  2003-2023 | Total Records Imported | | **468** | Total Records after Duplication | | | **240** | Included Studies | | **6** | Included Studies after CitationChaser | **2** |
| SR Screening 2003-2024 | Total Records Imported | | **557** | Total Records after Duplication | | | **251** | Included Studies | | **0** | Included Studies after CitationChaser | **0** |
| Total Number of Included Studies in the SR = 8 | | | | | | | | | | | | |
