# Supplementary material for: Health belief model-based educational interventions for knowledge, beliefs, and intentions on mammography: a systematic review
Source: BMC Womens Health. 2025 Dec 22;26:48. doi: 10.1186/s12905-025-04218-9 (PMC12836963; doi:10.1186/s12905-025-04218-9)
Supplement: Supplementary file 6 — Supplementary Material 6. [file 12905_2025_4218_MOESM6_ESM.docx]

**Supplementary Table 3:** Assessment of Bias Risk for RCTs Using the ROB 2 Tool.

**Study 1 (RCT)** - Title: The effectiveness of a nurse-delivered breast health promotion program on breast cancer screening behaviors in non-adherent Turkish women: A randomized controlled trial (Secginli, 2011)

| Domain | Description | Response |
| --- | --- | --- |
| 1.1 Was the allocation sequence random? | The women participated voluntarily and were unaware of their assigned group until the intervention began.  An introductory letter and questionnaire were sent to those identified from school guidance records.  The researcher reviewed the letters for eligibility, then called the women to confirm eligibility and explain the study. | Y |
| - 1. Was the allocation sequence concealed until participants were enrolled and assigned to interventions? |  | Y |
| - 1. Did baseline differences between intervention groups suggest a problem with the randomization process? | Eligible women were aged 41 and older, had not had an MMG in the past year, and had no history of BC. The study sample included mothers of students from a nearby primary school. No significant baseline differences were found between the two groups. | N |
|  | **LOW** | |
| 2.1 Were participants aware of their assigned intervention during the trial? | The women participated voluntarily and were unaware of their group assignments until the intervention.  An introductory letter and questionnaire were sent to women from the school guidance records. The researcher reviewed the letters for eligibility and later called the women to confirm eligibility and explain the study. | PY |
| 2.2 Were carers and people delivering the interventions aware of participants' assigned intervention during the trial? |  | PY |
| [If applicable:] If Y/PY/NI to 2.1 or 2.2: Were important non-protocol interventions balanced across intervention groups? | Participants in the intervention group received a breast health promotion program, while the control group received usual care. | PN |
| 2.4 [If applicable:] Were there failures in implementing the intervention that could have affected the outcome? | At the end of the sessions, the researcher reviewed key themes and encouraged participants to ask questions about mammography, CBE, and BSE.  Women practiced skills on a breast model with feedback from the researcher until they demonstrated proficiency. | PN |
| 2.5 [If applicable:] Was there non-adherence to the assigned intervention regimen that could have affected participants’ outcomes? | Twenty-six dropped out for a variety of reasons. Most participants who completed the study received the assigned intervention. | PN |
| 2.6 If N/PN/NI to 2.3, or Y/PY/NI to 2.4 or 2.5: Was an appropriate analysis used to estimate the effect of adhering to the intervention? | The data was analyzed using SPSS version 11. Descriptive statistics, chi-square tests, t-tests, Mann–Whitney U test, and repeated measures ANOVA assessed socio-demographic characteristics, changes in mammography, CBE, and BSE rates, as well as health beliefs and breast health knowledge over time. Adjusted odds ratios and 95% confidence intervals for practicing BSE at the 3- and 6-month follow-ups are reported, with significance evaluated at p< 0.05. | PY |
|  | **Some Concerns** | |
| 3.1 Were data for this outcome available for all, or nearly all, participants randomized? | All outcomes were available for all participants (190 women total: 97 in the intervention group, 93 in the control group). | Y |
| 3.2 If N/PN/NI to 3.1: Is there evidence that the result was not biased by missing outcome data? |  | NA |
| 3.3 If N/PN to 3.2: Could missingness in the outcome depend on its true value? |  | NA |
| 3.4 If Y/PY/NI to 3.3: Is it likely that missingness in the outcome depended on its true value? |  | NA |
|  | **LOW** | |
| 4.1 Was the method of measuring the outcome inappropriate? | Five pre-specified valid tools were used for data collection in this study. | N |
| 4.2 Could measurement or ascertainment of the outcome have differed between intervention groups? | All the participants in both groups have used the same measurement methods. | N |
| 4.3 If N/PN/NI to 4.1 and 4.2: Were outcome assessors aware of the intervention received by study participants? | The researcher reviewed the letters for eligibility, then called the women to reconfirm and explain the study. | PY |
| 4.4 If Y/PY/NI to 4.3: Could knowledge of the intervention received have influenced the assessment of the outcome? | There was no evidence that outcome assessment was influenced by knowledge of the intervention. Women in the intervention group were more likely to perform mammography, CBE, and BSE compared to the control group. | PN |
| 4.5 If Y/PY/NI to 4.4: Is it likely that assessment of the outcome was influenced by knowledge of the intervention received? |  | NA |
|  | **LOW** | |
| 5.1 Were the data that produced this result analyzed by a pre-specified analysis plan that was finalized before unblinded outcome data were available for analysis? | A predetermined analysis plan was established before collecting outcome data. Data were analyzed using SPSS version 16.0. Descriptive analyses summarized the variables, while Chi-square, t-test, and paired t-test were employed for data analysis, with a significance level set at α = 0.05. | Y |
| 5.2 ... multiple eligible outcome measurements (e.g., scales, definitions, time points) within the outcome domain? | All eligible reported results for the outcome domain correspond to all intended outcome measurements. | N |
| 5.3 ... multiple eligible analyses of the data? | All eligible reported results for the outcome measurement correspond to all intended analyses. | N |
|  | **LOW** | |
| LOW | | |

**Study 2 (RCT)** - Title: The effects of breast cancer educational intervention on knowledge and health beliefs of women 40 years and older, Isfahan, Iran (Rezaeian, 2014)

| Domain | Description | Response |
| --- | --- | --- |
| - 1. Was the allocation sequence random? | Out of 384 women aged 40 and older with no personal history of breast cancer, 290 agreed to participate in a study. They were randomly assigned to an intervention or control group. | Y |
| - 1. Was the allocation sequence concealed until participants were enrolled and assigned to interventions? |  | PY |
| - 1. Did baseline differences between intervention groups suggest a problem with the randomization process? | The intervention and control groups were similar in demographics, including education, marital status, employment, income, number of children, and awareness of BC. | N |
|  | **LOW** | |
| 2.1 Were participants aware of their assigned intervention during the trial? | Out of 290 women who initially agreed to participate in the education program, one from the intervention group dropped out due to a trip. Consequently, 144 women in the intervention group and 145 in the control group were included in the study. More evidence is needed to assess if the women knew their group assignment. | PY |
| 2.2 Were carers and people delivering the interventions aware of participants' assigned intervention during the trial? |  | PY |
| [If applicable:] If Y/PY/NI to 2.1 or 2.2: Were important non-protocol interventions balanced across intervention groups? | No evidence suggested that non-protocol interventions were evenly distributed between the intervention and control groups. | NI |
| 2.4 [If applicable:] Were there failures in implementing the intervention that could have affected the outcome? | There was no information regarding any failures in implementing the intervention that could have impacted the outcome. | NI |
| 2.5 [If applicable:] Was there non-adherence to the assigned intervention regimen that could have affected participants’ outcomes? | As evidenced by the study results, all participants who enrolled in it adhered to and completed the interventions. | PN |
| 2.6 If N/PN/NI to 2.3, or Y/PY/NI to 2.4 or 2.5: Was an appropriate analysis used to estimate the effect of adhering to the intervention? | Data were analyzed using SPSS version 16.0, employing descriptive analyses to summarize variables. Chi-square, t-test, and paired t-test were used, with significance set at α = 0.05. | PY |
|  | **LOW** | |
| 3.1 Were data for this outcome available for all, or nearly all, participants randomized? | All outcomes were available for all participants in the study. | Y |
| 3.2 If N/PN/NI to 3.1: Is there evidence that the result was not biased by missing outcome data? |  | NA |
| 3.3 If N/PN to 3.2: Could missingness in the outcome depend on its true value? |  | NA |
| 3.4 If Y/PY/NI to 3.3: Is it likely that missingness in the outcome depended on its true value? |  | NA |
|  | **LOW** | |
| 4.1 Was the method of measuring the outcome inappropriate? | Measurement methods:  1. Sociodemographic questions,  2. Knowledge about BC and  3. Questions about the HBM scale. | N |
| 4.2 Could measurement or ascertainment of the outcome have differed between intervention groups? | All the participants have the same measurement methods. | N |
| 4.3 If N/PN/NI to 4.1 and 4.2: Were outcome assessors aware of the intervention received by study participants? | There was no clear information to clarify that. | NI |
| 4.4 If Y/PY/NI to 4.3: Could knowledge of the intervention received have influenced the assessment of the outcome? | The study was a population-based controlled trial assessing the influence of outcome based on intervention knowledge.  Three hundred eighty-four women aged 40 and over were recruited via telephone interviews, with no personal history of breast cancer. Of these, 290 agreed to participate in an educational program, and participants were randomly assigned to either an intervention or control group. | PN |
| 4.5 If Y/PY/NI to 4.4: Is it likely that the assessment of the outcome was influenced by knowledge of the intervention received? |  | NA |
|  | **LOW** | |
| 5.1 Were the data that produced this result analyzed by a pre-specified analysis plan that was finalized before unblinded outcome data were available for analysis? | A predetermined analysis plan was established before outcome data collection. Data were analyzed using SPSS version 16.0 with descriptive analyses for summarizing variables. Chi-square, t-test, and paired t-test were employed, with significance set at α = 0.05. | Y |
| 5.2 ... multiple eligible outcome measurements (e.g., scales, definitions, time points) within the outcome domain? | All eligible reported results for the outcome domain correspond to all intended outcome measurements. | N |
| 5.3 ... multiple eligible analyses of the data? | All eligible reported results for the outcome measurement correspond to all intended analyses. | N |
|  | **LOW** | |
| LOW | | |

# **Study 3 (RCT)** - Title: Comparison of Two Different Educational Methods for Teachers’ Mammography Based on the Health Belief Model. (Heydari and Noroozi , 2015).

| Domain | Description | Response |
| --- | --- | --- |
| 1.1 Was the allocation sequence random? | Elementary school teachers were randomly grouped to minimize interaction and divided for multimedia education based on control variables and school criteria. | Y |
| - 1. Was the allocation sequence concealed until participants were enrolled and assigned to interventions? |  | NI |
| - 1. Did baseline differences between intervention groups suggest a problem with the randomization process? | The control variables and demographic characteristics were found to be similar between the two study groups. | N |
|  | **LOW** | |
| 2.1 Were participants aware of their assigned intervention during the trial? | The research staff conducted baseline surveys using the Computer Assisted Telephone Interviewing (CATI) system. The team included two senior investigators focused on cancer control and screening disparities in Asian American women, along with consultants in psychology, oncology nursing, and statistics. Interviewers had at least a bachelor’s degree in a health field and completed a 2-day intensive training. | PY |
| 2.2 Were carers and people delivering the interventions aware of participants' assigned intervention during the trial? |  | PY |
| [If applicable:] If Y/PY/NI to 2.1 or 2.2: Were important non-protocol interventions balanced across intervention groups? | The participants in both education and multimedia groups received similar training interventions. | PN |
| 2.4 [If applicable:] Were there failures in implementing the intervention that could have affected the outcome? | There was no information about any failures in implementing the intervention that could have affected the outcome. | NI |
| 2.5 [If applicable:] Was there non-adherence to the assigned intervention regimen that could have affected participants’ outcomes? | Both groups had the same dropout rate (5 participants), resulting in 60 participants each, exceeding the initial estimate of 52. Most participants who completed the study received the assigned intervention. | PN |
| 2.6 If N/PN/NI to 2.3, or Y/PY/NI to 2.4 or 2.5: Was an appropriate analysis used to estimate the effect of adhering to the intervention? | Data were analyzed with SPSS software (version 16.0) using descriptive statistics, Chi-square tests, t-tests, repeated measures ANOVA, Mann-Whitney U, and McNemar tests. | PY |
|  | **Some Concerns** | |
| 3.1 Were data for this outcome available for all, or nearly all, participants randomized? | All outcomes were available for all participants in the study. | Y |
| 3.2 If N/PN/NI to 3.1: Is there evidence that the result was not biased by missing outcome data? |  | NA |
| 3.3 If N/PN to 3.2: Could missingness in the outcome depend on its true value? |  | NA |
| 3.4 If Y/PY/NI to 3.3: Is it likely that missingness in the outcome depended on its true value? |  | NA |
|  | **LOW** | |
| 4.1 Was the method of measuring the outcome inappropriate? | Four valid data collection tools were used in this study:  Personal information, Knowledge of BC predisposing factors, HBM constructs, and Mammography questions. | N |
| 4.2 Could measurement or ascertainment of the outcome have differed between intervention groups? | All the participants in both groups have used the same measurement methods. | N |
| 4.3 If N/PN/NI to 4.1 and 4.2: Were outcome assessors aware of the intervention received by study participants? | In group education, subjects underwent training in two sessions, a week apart. In multimedia education, they received similar training through a CD and educational SMS on their cell phones, planned by researchers based on HBM. | PY |
| 4.4 If Y/PY/NI to 4.3: Could knowledge of the intervention received have influenced the assessment of the outcome? | Knowledge of intervention status might have influenced outcome assessment, but there's no evidence to suggest it did. Participants in both groups were chosen because they had no breast cancer or other cancers, no family history of breast cancer, and had not undergone a breast biopsy or mammography in the last three years. | PN |
| 4.5 If Y/PY/NI to 4.4: Is it likely that the assessment of the outcome was influenced by knowledge of the intervention received? |  | NA |
|  | **LOW** | |
| 5.1 Were the data that produced this result analysed by a pre-specified analysis plan that was finalized before unblinded outcome data were available for analysis? | A predetermined analysis plan was established before data collection. Data were analyzed using SPSS version 16.0 with descriptive statistics, Chi-square test, t-test, paired t-test, repeated measures ANOVA, Mann-Whitney U, and McNemar test. | Y |
| 5.2 ... multiple eligible outcome measurements (e.g., scales, definitions, time points) within the outcome domain? | All eligible reported results for the outcome domain correspond to all intended outcome measurements. | N |
| 5.3 ... multiple eligible analyses of the data? | All eligible reported results for the outcome measurement correspond to all intended analyses. | N |
|  | **LOW** | |
| LOW | | |

# **Study 4 (RCT)** - Title: Developing and Evaluating an Individually Tailored Intervention to Increase Mammography Adherence among Chinese American women. (Tsu-Yin, 2015)

| Domain | Description | Response |
| --- | --- | --- |
| - 1. Was the allocation sequence random? | A total of 193 participants enrolled in the study, with 96 in the intervention group and 97 in the control group. | Y |
| - 1. Was the allocation sequence concealed until participants were enrolled and assigned to interventions? |  | PY |
| - 1. Did baseline differences between intervention groups suggest a problem with the randomization process? | There were no significant differences between the intervention and control groups regarding demographics, health access, and key cognition and knowledge variables, indicating successful randomization. | N |
|  | **LOW** | |
| 2.1 Were participants aware of their assigned intervention during the trial? | Participants were randomly assigned to either the tailored telephone intervention or the control group. Those in the intervention received counseling tailored to their baseline interview results. | PY |
| 2.2 Were carers and people delivering the interventions aware of participants' assigned intervention during the trial? |  | PY |
| [If applicable:] If Y/PY/NI to 2.1 or 2.2: Were important non-protocol interventions balanced across intervention groups? | There was no evidence that non-protocol interventions were evenly distributed between the intervention and control groups. | NI |
| 2.4 [If applicable:] Were there failures in implementing the intervention that could have affected the outcome? | There was no information about any failures in implementing the intervention that could have affected the outcome. | NI |
| 2.5 [If applicable:] Was there non-adherence to the assigned intervention regimen that could have affected participants’ outcomes? | In the intervention group, 93 of 96 participants completed the study, while the control group had 96 of 97 participants complete the study. Both groups were similarly sized, randomly assigned, and had comparable demographics. A total of 189 participants were enrolled, surpassing the estimated 128. | PN |
| 2.6 If N/PN/NI to 2.3, or Y/PY/NI to 2.4 or 2.5: Was an appropriate analysis used to estimate the effect of adhering to the intervention? | Power analysis was based on intervention studies in non-Asian American women because of the limited availability of effect sizes for Chinese Americans. All participants will be non-compliant at baseline. A chi-square test with 64 participants per group shows a 71% power to detect a difference at a two-tailed alpha of 0.05, calculated using PASS software. | PY |
|  | **Some Concerns** | |
| 3.1 Were data for this outcome available for all, or nearly all, participants randomized? | All outcomes were available for all participants in the study. | Y |
| 3.2 If N/PN/NI to 3.1: Is there evidence that the result was not biased by missing outcome data? |  | NA |
| 3.3 If N/PN to 3.2: Could missingness in the outcome depend on its true value? |  | NA |
| 3.4 If Y/PY/NI to 3.3: Is it likely that missingness in the outcome depended on its true value? |  | NA |
|  | **LOW** | |
| 4.1 Was the method of measuring the outcome inappropriate? | Four measurement tools were used for data collection: Sociodemographic information, Knowledge Scale, Mammography Cognition Variables scale (Perceived Benefits, Barriers, and Self-Efficacy), and Participant satisfaction with the intervention scale. | N |
| 4.2 Could measurement or ascertainment of the outcome have differed between intervention groups? | All participants in both the interventional and control groups use the same measurement tools. | N |
| 4.3 If N/PN/NI to 4.1 and 4.2: Were outcome assessors aware of the intervention received by study participants? | There was no clear information to clarify that. | NI |
| 4.4 If Y/PY/NI to 4.3: Could knowledge of the intervention received have influenced the assessment of the outcome? | Knowledge of intervention status could have influenced the outcome assessment, but there is no evidence it did. In this randomized clinical trial, participants were assigned to either a tailored telephone intervention or a control condition. They were selected based on not having had a mammogram in the past 15 months and not having a BC diagnosis. | PN |
| 4.5 If Y/PY/NI to 4.4: Is it likely that the assessment of the outcome was influenced by knowledge of the intervention received? |  | NA |
|  | **LOW** | |
| 5.1 Were the data that produced this result analyzed by a pre-specified analysis plan that was finalized before unblinded outcome data were available for analysis? | A predefined analysis plan was established before obtaining the outcome data. Data were analyzed using SPSS 20, employing independent t-tests and paired t-tests for group comparisons, as well as a GLM Repeated-Measures test to assess changes within and between groups. | Y |
| 5.2 ... multiple eligible outcome measurements (e.g., scales, definitions, time points) within the outcome domain? | All eligible reported results for the outcome domain correspond to all intended outcome measurements. | N |
| 5.3 ... multiple eligible analyses of the data? | All eligible reported results for the outcome measurement correspond to all intended analyses. | N |
|  | **LOW** | |
| Some Concerns | | |

# **Study 5 (RCT)** - Title: Effect of Consultation on Adherence to Clinical Breast Examination and Mammography in Iranian Women: A Randomized Controlled Trial. (Mirmoammadi, 2018)

| Domain | Description | Response |
| --- | --- | --- |
| - 1. Was the allocation sequence random? | Eight healthcare centers in Hamadan City were randomly selected using cluster sampling and assigned to experimental or control groups. Twenty eligible women from each center participated in the study, but five women from each group were excluded due to absences. | Y |
| - 1. Was the allocation sequence concealed until participants were enrolled and assigned to interventions? |  | PY |
| - 1. Did baseline differences between intervention groups suggest a problem with the randomization process? | Demographic differences between the intervention and control groups were minimal, except that the experimental group had a lower education level, which was accounted for in the Analysis of Covariance Test. | PN |
|  | **LOW** | |
| 2.1 Were participants aware of their assigned intervention during the trial? | The population consisted of women in Hamadan, Iran, attending health centers for check-ups and cancer screenings.  Surveys, taking 15 minutes to complete, were administered independently.  Participants were informed about confidentiality and anonymity, with their data securely stored by the main researcher. More evidence is needed to assess whether women were aware of their group assignment. | PY |
| 2.2 Were carers and people delivering the interventions aware of participants' assigned intervention during the trial? |  | PY |
| [If applicable:] If Y/PY/NI to 2.1 or 2.2: Were important non-protocol interventions balanced across intervention groups? | There was no evidence that non-protocol interventions were equally distributed between the intervention and control groups. | NI |
| 2.4 [If applicable:] Were there failures in implementing the intervention that could have affected the outcome? | There was no information about any failures in implementing the intervention that could have affected the outcome. | NI |
| 2.5 [If applicable:] Was there non-adherence to the assigned intervention regimen that could have affected participants’ outcomes? | Out of 160 participants in the intervention group, five women were excluded due to absences and lack of follow-up, resulting in equal group sizes. Both groups were randomly assigned and had similar demographics, with a 6% attrition rate in each. | PN |
| 2.6 If N/PN/NI to 2.3, or Y/PY/NI to 2.4 or 2.5: Was an appropriate analysis used to estimate the effect of adhering to the intervention? | Data were analyzed using SPSS version 20. Independent and paired t-tests compared differences between and within groups, while the GLM repeated measurement test assessed changes within and between groups. | PY |
|  | **Some Concerns** | |
| 3.1 Were data for this outcome available for all, or nearly all, participants randomized? | All outcomes were available for all participants in the study. | Y |
| 3.2 If N/PN/NI to 3.1: Is there evidence that the result was not biased by missing outcome data? |  | NA |
| 3.3 If N/PN to 3.2: Could missingness in the outcome depend on its true value? |  | NA |
| 3.4 If Y/PY/NI to 3.3: Is it likely that missingness in the outcome depended on its true value? |  | NA |
|  | **LOW** | |
| 4.1 Was the method of measuring the outcome inappropriate? | The data collection tool was a questionnaire with four sections: Demographics, Knowledge of BCS, CBE, and mammography practices, and HBM constructs. | N |
| 4.2 Could measurement or ascertainment of the outcome have differed between intervention groups? | All participants in both the interventional and control groups use the same measurement tools. | N |
| 4.3 If N/PN/NI to 4.1 and 4.2: Were outcome assessors aware of the intervention received by study participants? | There was no clear information to clarify that. | NI |
| 4.4 If Y/PY/NI to 4.3: Could assessment of the outcome have been influenced by knowledge of the intervention received? | Intervention status knowledge might have influenced outcome assessment, but there's no evidence to suggest it did. The study involved women over 40 in Hamadan, Iran, attending health centers for services like routine check-ups and cancer screenings. Inclusion criteria included no detected breast cancer (BC) and no family history of BC in first-degree relatives. | PN |
| 4.5 If Y/PY/NI to 4.4: Is it likely that the assessment of the outcome was influenced by knowledge of the intervention received? |  | NA |
|  | **LOW** | |
| 5.1 Were the data that produced this result analysed in accordance with a pre-specified analysis plan that was finalized before unblinded outcome data were available for analysis? | A predetermined analysis plan was established before outcome data collection. Data were analyzed using SPSS 20, with independent and paired t-tests to compare group differences. The GLM Repeated Measures test assessed changes within and between groups. | Y |
| 5.2 ... multiple eligible outcome measurements (e.g., scales, definitions, time points) within the outcome domain? | All eligible reported results for the outcome domain correspond to all intended outcome measurements. | N |
| 5.3 ... multiple eligible analyses of the data? | All eligible reported results for the outcome measurement correspond to all intended analyses. | N |
|  | **LOW** | |
| LOW | | |
