# Supplementary material for: Health belief model-based educational interventions for knowledge, beliefs, and intentions on mammography: a systematic review
Source: BMC Womens Health. 2025 Dec 22;26:48. doi: 10.1186/s12905-025-04218-9 (PMC12836963; doi:10.1186/s12905-025-04218-9)
Supplement: Supplementary file 7 — Supplementary Material 7. [file 12905_2025_4218_MOESM7_ESM.docx]

# **Supplementary Table 4:** Assessment of Bias Risk for NRCTs Using the RoBANS 2 Tool.

**Study 1 (NRCT)** - Title: A culturally targeted intervention to promote breast cancer screening among low-income women in East Baltimore, Maryland (Garza, 2005).

|  | Domain | Details | Risk of Bias | Justification |
| --- | --- | --- | --- | --- |
| 1 | Comparability of the target group | Selection bias due to the selection of an inappropriate comparison target group | □ Low  □ High  □ Unclear | - No comparison group was included.  - The same population of 127 women was assessed before and after the intervention.  - Participants acted as their own comparison group by being evaluated three times during the intervention. |
| 2 | Target group selection | Selection bias due to inappropriate intervention or inappropriate selection of the exposure group or the patient group | □ Low  □ High  □ Unclear | The target group consisted of women aged 40 to 65 from low-income areas in East Baltimore who had never had a mammogram or had not had one in the last 5 years.  Community health workers recruited participants by going door-to-door, securing consent, and providing information about free MMG screenings at Johns Hopkins Hospital for those who chose not to participate in the study. |
| 3 | Confounders | Selection bias due to inappropriate confounder confirmation and consideration | □ Low  □ High  □ Unclear | Exclusion was not possible, but the data were revised during analysis.  The study design involved multiple participant tests, which could introduce test-retest bias; to minimize this, the questions were rearranged.  Concerns about carryover effects arose due to non-random intervention stages, but as interventions became more potent at each stage, it is unlikely that carryover effects occurred. |
| 4 | Measurement of intervention/  exposure | Performance bias due to inappropriate intervention or inappropriate exposure measurement | □ Low  □ High  □ Unclear | A modified time series design included four stages: home visits, brochure distribution, an educational session at a local church, and scheduling appointments at a breast clinic. Measurements included sociodemographic questions, knowledge about breast cancer, and the CHBM scale. |
| 5 | Blinding of assessors | Detection bias due to inappropriate blinding of assessors | □ Low  □ High  □ Unclear | No blinding was done for outcome assessors, but its absence is not thought to have impacted on the results. |
| 6 | Outcome assessment | Detection bias due to inappropriate outcome assessment methods | □ Low  □ High  □ Unclear | - Outcomes were assessed using reliable and valid tools.  - Changes in knowledge about BC and mammography were measured by the percentage of correct answers at each stage.  - A similar analysis was done for beliefs about breast cancer and mammography. |
| 7 | Incomplete outcome data | Attrition bias due to inappropriate handling of incomplete data | □ Low  □ High  □ Unclear | A total of 127 women were recruited for the study, with 118 (93%) having never had a mammogram. Most participants (n=119) completed all three stages, while eight did not finish due to one death and seven with incomplete data; these participants were similar to those who completed the study. |
| 8 | Selective outcome reporting | Reporting bias due to selective outcome reporting | □ Low  □ High  □ Unclear | All primary and secondary outcomes specified in the predetermined protocol were clearly described as intended. |

**Study 2 (NRCT)** - Title: Development and evaluation of a culturally tailored educational video: changing breast cancer-related behaviors in Chinese women. (Wang, 2008).

|  | Domain | Details | Risk of Bias | Justification |
| --- | --- | --- | --- | --- |
| 1 | Comparability of the target group | Selection bias due to the selection of an inappropriate comparison target group | □ Low  □ High  □ Unclear | - No comparison group was included.  - The population group remains the same before and after the intervention (44 women participated in both pretests and posttests). |
| 2 | Target group selection | Selection bias due to inappropriate intervention or inappropriate selection of the exposure group or the patient group | □ Low  □ High  □ Unclear | The target group was recruited systematically, and data were collected prospectively.  Initially, 225 women were approached, with 90 meeting eligibility criteria. Among these, 58% (52) consented to participate, while eight were excluded. The final evaluation sample included 44 women for pretests and posttests. |
| 3 | Confounders | Selection bias due to inappropriate confounder confirmation and consideration | □ Low  □ High  □ Unclear | Exclusion was not possible; however, data were revised during analysis.  All respondents recalled the characters, plot, content, and physician's recommendations of the video.  A repeated measures ANOVA was conducted, retaining a significance level of p ≤ .05 without adjustment, using SPSS version 12.0 for all analyses. |
| 4 | Measurement of intervention/  exposure | Performance bias due to inappropriate intervention or inappropriate exposure measurement | □ Low  □ High  □ Unclear | The research comprised three stages:  (a) a formative phase to identify themes and video format,  (b) a production phase with local Chinese communities and video companies, and  (c) a quantitative phase assessing video acceptability and effectiveness through a pretest/posttest design.  Validated questionnaires for mammography intentions and barriers, with baseline measures re-evaluated to track changes after the intervention. |
| 5 | Blinding of assessors | Detection bias due to inappropriate blinding of assessors | □ Low  □ High  □ Unclear | No blinding was done for outcome assessors, but this likely did not affect the outcomes. |
| 6 | Outcome assessment | Detection bias due to inappropriate outcome assessment methods | □ Low  □ High  □ Unclear | Outcomes were evaluated by using reliable tools.  The study focused on 44 Chinese women to assess changes in screening intention, knowledge, health beliefs, and cultural views. The McNemar test analyzed changes in screening intention, while paired t-tests assessed changes in continuous variables before & after the study. |
| 7 | Incomplete outcome data | Attrition bias due to inappropriate handling of incomplete data | □ Low  □ High  □ Unclear | Fifty-two women took part in the baseline assessment, but 6 (12%) did not complete the follow-up. After excluding 2 participants with prior mammograms, the final sample was 44 women for both pretests and posttests. Missing values for continuous baseline variables were replaced with the overall mean, but those from loss of contact or refusal during follow-up were not addressed. |
| 8 | Selective outcome reporting | Reporting bias due to selective outcome reporting | □ Low  □ High  □ Unclear | All outcomes in the predetermined protocol were described as planned. |

**Study 3 (NRCT)** - Title: Interventional Education Methods for Increasing Women's Participation in Breast Cancer Screening Programs. (Seven, 2015).

|  | Domain | Details | Risk of Bias | Justification |
| --- | --- | --- | --- | --- |
| 1 | Comparability of the target group | Selection bias due to the selection of an inappropriate comparison target group | □ Low  □ High  □ Unclear | The study included three intervention groups, each using a different educational method.  Researchers used block randomization to obtain and sort the contact information of participants, followed by home visits to assess their eligibility for the study. |
| 2 | Target group selection | Selection bias due to inappropriate intervention or inappropriate selection of the exposure group or patient group | □ Low  □ High  □ Unclear | The target groups were recruited systematically, and data were collected prospectively.  Convenience sampling selected Ankara, Trabzon, and Malatya for the study, using a two-stage stratified method to sample 551 women aged 50 to 69: 174 from Ankara, 237 from Trabzon, and 140 from Malatya. The study had a 2.5% error rate and a 95% confidence interval. |
| 3 | Confounders | Selection bias due to inappropriate confounder confirmation and consideration | □ Low  □ High  □ Unclear | Exclusion was not possible, but the data were revised during analysis. Univariate and multivariate logistic regression analyses were conducted to examine factors influencing participants' screening rates after their home visit. |
| 4 | Measurement of intervention/  exposure | Performance bias due to inappropriate intervention or inappropriate exposure measurement | □ Low  □ High  □ Unclear | - The study's brochures were created after a literature review, assessment of existing materials, and expert input.  - Two brochures targeted women and their spouses, focusing on breast cancer awareness and early detection.  - Measurements included: Participant Description Questionnaire, Knowledge Evaluation Form, CHBMS, and Reason Identification Form. |
| 5 | Blinding of assessors | Detection bias due to inappropriate blinding of assessors | □ Low  □ High  □ Unclear | - Outcome assessors were not blinded, but this likely did not affect the results. |
| 6 | Outcome assessment | Detection bias due to inappropriate outcome assessment methods | □ Low  □ High  □ Unclear | Participants' outcomes were assessed using reliable and valid tools. Data analysis was performed with SPSS version 15.0, including descriptive statistics, chi-squared test, Kruskal-Wallis test, Mann-Whitney U test, paired t-test, and regression analyses. A p-value of less than 0.05 was deemed statistically significant. |
| 7 | Incomplete outcome data | Attrition bias due to inappropriate handling of incomplete data | □ Low  □ High  □ Unclear | All participants met the study requirements and completed the study without any withdrawals. |
| 8 | Selective outcome reporting | Reporting bias due to selective outcome reporting | □ Low  □ High  □ Unclear | All outcomes outlined in the predetermined protocol were described as intended. |
