# Supplementary material for: Health belief model-based educational interventions for knowledge, beliefs, and intentions on mammography: a systematic review
Source: BMC Womens Health. 2025 Dec 22;26:48. doi: 10.1186/s12905-025-04218-9 (PMC12836963; doi:10.1186/s12905-025-04218-9)
Supplement: Supplementary file 8 — Supplementary Material 8. [file 12905_2025_4218_MOESM8_ESM.docx]

**Supplementary Table 5:** Summary of Bias Risk Assessment Using RoB 2 for RCTs

| Study Author | | Experimental | | | | Comparator | | Outcome | | | | D1^a^ | | D2^b^ | | D3^c^ | D4^d^ | | | | D5^e^ | Overall |
| --- | --- | --- | --- | --- | --- | --- | --- | --- | --- | --- | --- | --- | --- | --- | --- | --- | --- | --- | --- | --- | --- | --- |
| Secginli & Nahcivan /  2011 | | Intervention | | | | Intervention | | Knowledge, Belief, and Intention | | | | \|  \| \| --- \| | | \|  \| \| --- \| | | \|  \| \| --- \| | \|  \| \| --- \| | | | | \|  \| \| --- \| | \|  \| \| --- \| |
| Rezaeian/  2014 | | Intervention | | | | Control | | Knowledge, Belief, and Intention | | | | \|  \| \| --- \| | |  | |  |  | | | |  |  |
| Heydari & Noroozi/  2015 | | Intervention | | | | Intervention | | Knowledge, Belief, and Intention | | | | \|  \| \| --- \| | |  | |  |  | | | |  |  |
| Wu & Lin/  2015 | | Intervention | | | | Control | | Knowledge, Belief, and Intention | | | | \|  \| \| --- \| | |  | |  |  | | | |  |  |
| Mirmoammadi/ 2018 | | Intervention | | | | Control | | Knowledge, Belief, and Intention | | | | \|  \| \| --- \| | |  | |  |  | | | |  |  |
| Key | | | | | | | | | | | | | | | | | | | | | | |
|  | | | Low risk | | | |  | | | Some concerns | | | |  | | | | | High risk | | | |
| a | Randomization process | | | **b** | Deviations from the intended interventions | | | | **c** | | Missing outcome data | | **d** | | Measurement of the outcome | | | **e** | | Selection of the reported result | | |
