# Supplementary material for: Health belief model-based educational interventions for knowledge, beliefs, and intentions on mammography: a systematic review
Source: BMC Womens Health. 2025 Dec 22;26:48. doi: 10.1186/s12905-025-04218-9 (PMC12836963; doi:10.1186/s12905-025-04218-9)
Supplement: Supplementary file 9 — Supplementary Material 9. [file 12905_2025_4218_MOESM9_ESM.docx]

# **Supplementary Table 6:** Summary Bias Risk Assessment Using RoBANS 2 for NRCTs

| Study Author | Comparability of the target group | Target group selection | Confounders | Measurement of intervention  /exposure | Blinding of assessors | Outcome assessment | Incomplete outcome data | Selective outcome reporting | Overall |
| --- | --- | --- | --- | --- | --- | --- | --- | --- | --- |
| Garza  2005 | Low* | Low | Low | Low | Unclear* | Low | Low | Low | **Low** |
| Wang  2008 | Low | Low | Low | Low | Unclear | Low | Low | Low | **Low** |
| Seven  2015 | Low | Low | Low | Low | Unclear | Low | Low | Low | **Low** |

KEY - ***Risk of Bias**: Low Risk of Bias, High Risk of Bias, Unclear Risk of Bias.
