# Supplementary material for: Health belief model-based educational interventions for knowledge, beliefs, and intentions on mammography: a systematic review
Source: BMC Womens Health. 2025 Dec 22;26:48. doi: 10.1186/s12905-025-04218-9 (PMC12836963; doi:10.1186/s12905-025-04218-9)
Supplement: Supplementary file 10 — Supplementary Material 10. [file 12905_2025_4218_MOESM10_ESM.docx]

**Supplementary Table 7**: Included Studies Characteristics

| **Author,**  **Year/ Country** | **Journal** | **Participant** | **Setting** | **Education Level** | **Marital Status** |
| --- | --- | --- | --- | --- | --- |
| **Garza, 2005/**  **U.S.** | Cancer Control Journal | 127 Women  Immigrant African American  Aged from 40 to 65 | Community | Less than High School: 18 High School: 96  Some College: 7  College: 5 | Married:8,  Widowed:12  Divorced/ Separated:13  Never Married: 93 |
| **Wang, 2008/**  **U.S.** | Health education & behavior Journal | 43 Women  Immigrant Chinese Americans  Aged above 40 | Community | College:22  High School or Less:21 | NI* |
| **Secginli & Nahcivan, 2011/**  **Turkey** | International Journal of Nursing Studies | 190 Women  Turkish  Aged above 41 | Health Care Centers | 54% attended school  1-8 years,  46% attended school  more than 9 years | Married:92% Not-Married:8% |
| **Rezaeian, 2014/**  **Iran** | Journal of education and health promotion | 289 Women  Iranian  Aged above 40 | Health Care Center | Illiterate: 85  Elementary: 72  Under Diploma: 49  Diploma: 59  University: 25 | Married:250  Widowed:35 Divorced/ Separated:5 |
| **Seven, 2015/**  **Turkey** | Journal of Cancer Education | 327 Women  Turkish  Aged above 40 | Community | Literate: 83 Primary school graduate:174 High school graduate:54 University and higher:16 | Married: 255  Single: 6  Divorced/Widowed:66 |
| **Heydari & Noroozi,**  **2015/**  **Iran** | Asian Pacific Journal of Cancer Prevention | 120 Women  Iranian  Aged from 50 to 69 | Community | Diploma:52 Bachelor or Master:88 | Married:113  Single:3  Divorced/Widowed:4 |
| **Wu & Lin,**  **2015/**  **U.S.** | Cancer Nursing Journal | 193 Women  Immigrant Chinese Americans  Aged above 41 | School | Less than high school:84.5% More than high school:15.5% | Married:78%  Not Married:22% |
| **Mirmoammadi, 2018/**  **Iran** | Asian Pacific Journal of Cancer Prevention | 150 Women  Iranian  Aged above 40 | Health Care Centers | Illiterate:19 Primary:68  Secondary:33  High School: 14 Tertiary:17 | Single:12 Married:138 |

**Note:** NI- No Information.
