# Supplementary material for: Health belief model-based educational interventions for knowledge, beliefs, and intentions on mammography: a systematic review
Source: BMC Womens Health. 2025 Dec 22;26:48. doi: 10.1186/s12905-025-04218-9 (PMC12836963; doi:10.1186/s12905-025-04218-9)
Supplement: Supplementary file 11 — Supplementary Material 11. [file 12905_2025_4218_MOESM11_ESM.docx]

# **Supplementary Table 8**: Study Outcomes and Limitations of Included Studies.

| Author/  Year | Outcome Measures | Outcomes | Limitation |
| --- | --- | --- | --- |
| Garza/  2005 | 1.Sociodemographic Characteristics, 2. Knowledge about BC & Mammograms 3. CHBMS. | - There was a significant increase in knowledge about BC and mammography between stages 2 and 3 (P < 0.0001).  Mean scores did not change significantly for:  - Perceived susceptibility: 2.88 to 2.91 (P = 0.79).  - Perceived benefits: 3.58 to 3.68 (P = 0.32).  - However, perceived barriers decreased significantly from 2.24 to 2.06 (P < 0.0001).  - 77% of participants had an income of less than $6,000.  49.6% of those attending all intervention stages completed no-cost BCS and showed greater knowledge (P = 0.047).  - 58% were uninsured; 53.2% of them completed no-cost screenings, versus 10% with private insurance (P=.0089).  The only significant predictor was private insurance, with an odds ratio of 0.12 (P = 0.0486; 95% CI: 0.014–0.987). | - The small sample size and convenience sampling limit the generalizability to women in extreme poverty.  - We lacked data from non-participants.  - Multiple testing may introduce bias, and there was no comparison group.  - The lack of long-term follow-up raises questions about the sustainability of the intervention effects. |
| Wang/  2008 | 1.Sociodemographic and Medical Factors 2. Screening Intention to MMG Scale 3. Knowledge of screening guidelines BC, and the advantages of MMG 4. Cultural Views of Cancer &Healthcare 5. Health Beliefs (The Chinese MMG Screening Beliefs Questionnaire). 6.EnglishProficiency  7.ProcessEvaluation | Significant increases from baseline to follow-up:  -Knowledge: 7.36 ± 1.88 to 8.43 ± 1.40 (P = 0.001)  -Perceived susceptibility: 10.25±2.22 to 12.24±2.19 P=.00)  -Perceived benefits: 23.72 ± 1.80 to 27.93 ± 3.31 (P=.08)  -Perceived barriers: 44.65 ± 8.42 to 31.09 ± 8.88 (P=.00)  -Perceived seriousness: 26.16 ± 2.17 to 27.66 ± 4.12  (P= .08, not significant).  - Women with incomes over $20,000 had minor increases in knowledge and seriousness scores (Δ = 0.34 and Δ = -0.53, respectively), while low-income women showed greater improvements in perceived benefits (Δ = 5.24) compared to high-income women (Δ = 2.79).  - BCS intentions increased from 37% at baseline to 88% at follow-up (P < .0001). | - A participation rate of 58% raises concerns about bias and limits the findings' generalizability.  - The small voluntary focus group and evaluation sample size hindered multivariate analysis and independent associations.  - Due to time and funding constraints prevented the inclusion of actual MMG utilization or a control group, limiting conclusions about the video's effectiveness. |
| Author/  Year | **Outcome Measures** | **Outcomes** | **Limitation** |
| Secginli & Nahcivan  /2011 | 1. Demographic Data Questionnaire.  2. Follow-up questionnaire for (BSE, CBE, and Mammogram). 3. Breast Self-Examination Proficiency Rating Instrument. 4. CHBMS. 5. The Breast Health Knowledge (BHK) form. | Before the program, the average knowledge level of the intervention group (8.85 ± 2.40) was similar to the control group (8.98 ± 2.45). After the intervention, the intervention group showed a significant increase in breast health knowledge (df = 3; F = 789.86; p < 0.001) and improvements in:  - Perceived susceptibility: 12.8 (3.6) to 16 (2.9)  - Perceived benefits: 20.8 (2.8) to 22.2 (1.9)  - Perceived barriers: 17.24 (3.5) to 23.9 (2.9)  - Self-efficacy: 31.8 (6.3) to 39.9 (2.9)  The control group had changes in self-efficacy and perceived barriers, with a decrease in perceived benefits.  At six months, the intervention group scored significantly higher on health belief scales (P < 0.05), except for barriers to mammography (P = 0.116).  Mammography usage rates were similar: 15.5% for the intervention group & 9.7% for the control group (P=0.23). | - Findings may not apply to a larger population due to a small, regional sample of women.  - The brief intervention and six-month follow-up limit long-term conclusions.  - Self-reported data for mammography and clinical breast exams is a limitation.  - Cultural impacts on health behaviors were not assessed, highlighting a drawback of the HBM. |
| Rezaeian /2014 | 1.Sociodemographic Questions, 2. BC Knowledge  3. CHBMS. | An independent t-test showed that before the intervention, knowledge and health beliefs scores were similar between the two groups, except for perceived severity and health motivation.  After four weeks of the educational intervention, the intervention group showed significant improvements:  - Knowledge: 4.46 ± 3.12 to 10.12 ± 1.38, P = 0.002  -Perceived susceptibility: 9.64±2.58 to 11.67±1.93, P<.001  -Perceived severity: 23.08±4.61 to 27.03±4.18, P<.001  -Perceived benefits: 20.34±2.78 to 25.28±2.96, P<.001  -Health motivation: 23.97±4.72 to 26.69±4.43, P<.001  - Self-efficacy: 11.35 ± 3.64 to 15.77 ± 2.78, P < 0.001  - Perceived barriers decreased from 25.68±6.29 to 18.79± 5.43, P<.001  According to the HBM, women who perceive more benefits and fewer barriers to MMG are more likely to participate in screening. | - Evaluated women’s knowledge and beliefs about MMG four weeks post-educational intervention, limiting our assessment of actual practices.  - Focus on the HBM excluded social factors like norms.  - Suggest future research integrate the TPB with HBM for greater insight. |

| Author/  Year | Outcome Measures | Outcomes | Limitation |
| --- | --- | --- | --- |
| Seven/  2015 | 1. Participant Description Questionnaire,  2. Knowledge Evaluation Form,  3. CHBMS for BCS  4. Reason Identification Form. | There was no significant difference in screening rates among the three education methods (P = 0.067).  However, group education led to a higher screening MMG rate compared to individual education (P = 0.034).  Mean knowledge scores increased significantly for all groups post-education (P < 0.001).  Health motivation was the only significant change in CHBM subscales.  Multivariate logistic regression showed that education method and post-education knowledge scores significantly impacted screening decisions, with group education resulting in a screening rate 0.6 times higher than individual education. | - There was no system to ensure spouses received the brochure. While screening rates for women who received individual education were similar regardless of the brochure, its effectiveness is unclear.  - Women who received both education and the brochure weren't asked if their spouses understood it, complicating outcome comparisons. |
| Heydari & Noroozi /  2015 | 1.Sociodemo-graphic characteristics questionnaire, 2. Knowledge about the BC questionnaire,  3. The HBM scale questionnaire,  4. Questions related to Mammography. | - The group education results indicated significant changes in pre-test and post-test scores for knowledge, health motivation, perceived benefits, and barriers, with barriers decreasing (P < 0.006).  - In the Multimedia group, knowledge increased (P < 0.001) and perceived barriers decreased (P = 0.007) after three months, but no significant changes were found in perceived susceptibility, severity, or health motivation (P > 0.05).  - There were **no significant differences in scores between the two groups** (P > 0.05).  - Health motivation and perceived benefits were higher in the education group.  - After education, 93.33% of the education group and 83.33% of the multimedia group intended to undergo mammography (P= 0.088).  - Three months later, 80% of the education group and 55% of the multimedia group had completed the procedure (P= 0.003). | - The follow-up period was short; a duration of six months might have increased the number of individuals receiving MMG.  - Self-reported health beliefs and knowledge could result in overestimation. |

| Author/  Year | Outcome Measures | Outcomes | Limitation |
| --- | --- | --- | --- |
| Wu /2015 | 1.Sociodemographic information.  2. Knowledge Scale, 3.Mammography-Related Cognition Variables (Perceived Benefits, Barriers, and Self-Efficacy) scale. 4. Participant’s satisfaction with the intervention scale. | There were no significant differences between the intervention and control groups in mean scores for:  - Knowledge: 2.53 vs. 2.22 (P = 0.33)  - Perceived benefits: 3.20 vs. 3.28 (P = 0.20)  - Perceived barriers: 2.47 vs. 2.49 (P = 0.73)  - Self-efficacy: 2.79 vs. 2.76 (P = 0.55)  - After four months, 40% of the intervention group and 33% of the control group received mammograms, with no significant difference (χ²(1) = 1.81, P = ns).  - Among insured women, 56% in the intervention group participated versus 34%, a 22% difference.  - Older women (65+) experienced a greater effect, with 51% in the intervention group versus 25% in the control group, resulting in a 26% difference. | - The study relied on self-reported mammography data, which may over-report due to social desirability bias.  - Findings may not be applicable to Chinese American women with different demographics.  - Potential bias exists from self-selection, and we lack data on non-respondents to evaluate response bias. |
| Mirmoammadi/  2018 | 1. Demographic information,  2. Knowledge of BCS, 3. CBE and mammography practices,  4. HBM constructs. | The two groups showed no significant differences in knowledge and CHBMS constructs before the intervention. However, after three months of education, the intervention group made significant progress:  - Knowledge: 45.09 to 73.75 (P<0.001)  - Perceived susceptibility: 49.06 to 52.53 (P=0.148)  - Perceived severity: 63.21 to 67.56 (P=0.554)  - Perceived benefits: 77.07 to 86.58 (P<0.001)  - Health motivation: 79.8 to 86.69 (P<0.01)  - Self-confidence: 57.46 to 81.90 (P<0.001)  - Perceived barriers: 58.45 to 50.04 (P<0.01)  Mammography screening rates rose from 26.7% to 49.3%.  The main reasons for not screening were cost (49%), lack of awareness (21%), and perceived lack of need (12%).  Key information sources included radio and TV (44%), friends and relatives (34%), and healthcare providers (13%). | - The small sample size may limit the study’s generalizability, and  - The short follow-up period is due to cost and time constraints.  - Cannot guarantee that participants did not receive BCS training from other sources despite our request to avoid other educational programs. |
